# Puerarin Prevents Acute Liver Injury *via* Inhibiting Inflammatory Responses and ZEB2 Expression

**DOI:** 10.3389/fphar.2021.727916

**Published:** 2021-08-06

**Authors:** Junfa Yang, Maomao Wu, Hui Fang, Yue Su, Lingling Zhang, Huan Zhou

**Affiliations:** ^1^ Key Laboratory of Anti-inflammatory and Immune Medicine, Ministry of Education, Institute of Clinical Pharmacology, Anhui Medical University, Hefei, China; ^2^ School of Pharmacy, Anhui Medical University, Hefei, China; ^3^ Department of Pharmacy, Anhui Chest Hospital, Hefei, China; ^4^ Hangzhou Normal University Affiliated Hospital, Hangzhou, China; ^5^ Institute of Clinical Trial, The First Affiliated Hospital of Bengbu Medical College, Bengbu, China; ^6^ School of Public Basic, Bengbu Medical College, Bengbu, China

**Keywords:** puerarin, ali, inflammatory responses, ZEB2, proinflammatory

## Abstract

Puerarin, an isoflavone component extracted from herb radix puerariae, is widely used in China in the treatment of immune diseases and inflammation. Previous studies have demonstrated that puerarin prevented acute lung injury by regulating inflammatory responses. However, the effect of puerarin on acute liver injury (ALI) was unclear. The purpose of this study was to explore the beneficial effects of puerarin when applied to ALI. We found that puerarin inhibited liver injury and inflammatory cell infiltration in lipopolysaccharide (LPS)/D-galactose (D-Gal)-induced acute liver failure and the liver pro-inflammatory cytokines interleukin (IL)-1β, IL-6, and tumor necrosis factor-alpha (TNF-α) in liver tissues with ALI and LPS-induced L-02 cells but upregulated the expression level of zinc finger E-box-binding homeobox 2 (ZEB2). Significantly, the results of this study showed that the inhibition of liver pro-inflammatory cytokine (IL-1β, IL-6, and TNF-α) production in LPS-induced L-02 cells was caused by ZEB2 overexpression. However, knocking down ZEB2 promoted LPS-mediated secretion of liver pro-inflammatory cytokines in L-02 cells. Additional experiments showed that puerarin inhibited the activation of the NF-κB signaling pathway by elevating ZEB2 expression in L-02 cells. In summary, puerarin most likely prevented activation of the pro-inflammatory factors and reduced LPS/D-Gal-induced liver injury by enhancing the ZEB2 expression level and, consequently, blocking activation of the NF-κB signaling pathway in the liver.

## Introduction

As a key organ in the maintenance of energy balance, metabolic homeostasis, immune regulation, protein synthesis, and detoxification (Taub, 2004), the liver plays a leading role in drug metabolism and defense against infections, and is usually damaged as a result of drug overdose and/or severe infections (Lofthus et al., 2012). Acute liver injury (ALI) has a high rate of global morbidity and mortality, which may lead to chronic liver diseases, or even liver failure (Lofthus et al., 2012). Alcohol, drugs, toxins, chronic autoimmune hepatitis, and metabolic diseases have all been demonstrated to cause ALI (Wang Y.-y. et al., 2018; Shi et al., 2018; Yang et al., 2021). Furthermore, other studies also have shown that inflammatory responses play a critical role in ALI pathogenesis (Yang et al., 2020). Significantly, inhibiting the secretion of inflammatory cytokines might be a potential strategy for blocking ALI (Rivera et al., 2020). In addition, the results of recent studies have shown that the pathogenesis of ALI can be blocked by the inhibition of inflammatory responses (Starkey Lewis et al., 2020). In summary, anti-inflammatory therapies are effective in treating ALI (Chen et al., 2020; Zhang et al., 2020). It is, however, necessary to clarify the underlying mechanism of effective targets for ALI caused by sepsis in view of the fact that there is no current effective treatment for ALI (Bae et al., 2018).

ZEB2, part of zinc finger E homeobox-binding protein family, acts mainly as a transcription repressor of the SMAD protein (Scott and Omilusik, 2019). A great deal of evidence has shown that an abnormal expression level of ZEB2 is involved in several liver diseases, including liver cancer and hepatic fibrosis (Katoh and Katoh, 2009; Yang et al., 2017; Zhang et al., 2019). Recently, several investigations have shown that the production of inflammatory cytokines and the epithelial-to-mesenchymal transition (EMT) can be mediated by ZEB2 (Ding et al., 2018; Yang et al., 2021). In addition, activated nuclear factor kappa-light-chain-enhancer of activated B cells (NF-κB) enters the nucleus and induces expression of the numerous genes involved in cell adhesion, innate and adaptive immune regulation, anti-apoptotic mechanisms, and inflammatory responses (Vallabhapurapu and Karin, 2009; Hayden and Ghosh, 2012). A number of studies have shown that various natural herbs can play a role in suppressing anti-inflammatory responses by inhibiting the NF-κB signaling pathway (Remppis et al., 2010; Liang et al., 2014). Notably, Barbu et al. (2012) demonstrated that knockdown of ZEB2 can promote the LPS-mediated NF-κB response, although high-affinity IgE receptor (FcεRI)-induced NF-κB activation was inhibited (Barbu et al., 2012). Hence, investigating how ZEB2 participates in the inflammatory response may expedite the discovery of new therapeutic targets and efficacious treatment strategies for ALI.

Puerarin is a natural flavonoid compound that isolated from the traditional Chinese herb radix puerariae (Wu et al., 2007). Nowadays, puerarin has received increasing attention for its beneficial effects on inflammation (Xiao et al., 2011; Yao et al., 2012). A previous study revealed that puerarin plays a crucial role in a variety of pharmacological properties, including anti-oxidant, anti-inflammatory, cardioprotective, anticancer, and antidiabetic properties (Li et al., 2014; Yang et al., 2016). For example, Zhang et al. found that osteoclast formation and bone loss induced by LPS was inhibited puerarin (Zhang et al., 2016). Meanwhile, inflammatory responses and apoptosis were also inhibited by puerarin in LPS-stimulated cardiomyocytes and acute lung injury (Yuan et al., 2016; Wang X. et al., 2018). More importantly, researchers reported that puerarin inhibits iNOS, COX-2 and CRP expression via suppression of NF-κB activation in LPS-induced RAW264.7 macrophage cells (Hu et al., 2011). However, whether puerarin has a protective effect against the inflammatory response in ALI remains unclear.

In this study, we have confirmed that puerarin inhibits the expression of IL-1β, TNF-α, and IL-6, thereby attenuating the LPS-induced inflammatory response of L-02 cells and LPS/D-Gal-induced ALI. Furthermore, the expression of ZEB2 was reduced in ALI, while it was enhanced by puerarin. Mechanistic studies have shown puerarin regulates ZEB2 via NF-κB signaling in ALI to attenuate the inflammatory response.

## Materials and Methods

### Materials and Reagents

Puerarin (purity >98%, provided by Anhui Medical University), LPS, and D-Gal (Aibsin Biotechnology Co., Ltd., Shanghai, China) were used without further purification. β-Actin monoclonal antibody was from Bioworld, China. Phospho-p65 and Phospho-IκBα polyclonal antibodies were from Cell Signaling Technology (Danvers, MA, United States ). Human IL-1β, TNF-α, and IL-6 enzyme-linked immunosorbent assay (ELISA) kits were provided by Nanjing Fcmacs Biotechnology Co., Ltd. (Jiangsu, China). The polyclonal antibodies for IL-1β, TNF-α, and IL-6 were provided by Bioss (Beijing, China). Human ZEB2 was purchased Abcam, United States . The AST and ALT assay kits were from Nanjing Jiancheng Biology Engineering Institute PeproTech (Nanjing China). Dulbecco’s Modified Eagle Medium (DMEM) was supplied by Invitrogen, Thermo Fisher Scientific, Inc. (Waltham, MA, United States ). The antibiotic-antimitotic reagent was supplied by Invitrogen, and the human ELISA kits were supplied by Hangzhou MultiSciences (Lianke) Biotech, Co., Ltd. (Hangzhou, China). β-Actin (BS6007M, 1:1,000). Phospho-p65 (3,033, 1:1,000). Phospho-IκBα polyclonal (2,859, 1:1,000). Human IL-1β, TNF-α, and IL-6 (bs-0812R, bs-0781R and bs-16610R, 1:500). ZEB2 (ab223688, 1:500).

### Animals and Treatment

Male C57BL/6 mice (6–8 weeks old) were obtained from the Experimental Animal Center of Anhui Medical University, fed with the standard laboratory diet and water ad libitum, and kept in a controlled environment at 20–25°C, 50 ± 5% relative humidity, and a 12 h dark/light cycle. The research protocol was approved by Anhui Medical University’s local animal care committee. LPS/D-Gal-induced ALI in animals was induced as previously described (Yang et al., 2021). In brief, ALI was induced by intraperitoneal injection of D-Gal (700 mg/kg) and LPS (20 μg/kg) in 200 μl. On the same time, the different concentrations of puerarin (25 mg/kg, 50 mg/kg and 100 mg/kg) were used by gavage. Phosphate buffered saline (PBS) was used for the sham treatment. All experiments were carried out in accordance with the ethical guidelines of nursing institutions using laboratory animals at Anhui Medical University. The procedures involving animals were carried out in accordance with NIH guidelines and were approved by the Animal Care and Use Committee (No. LLSC20150348).

### Serum Aminotransferase Activity

Following collection, the blood samples were centrifugated at 5,000 rpm for 10 min. Commercially available kits were then used to assay the activity of serum aspartate aminotransferase (alanine transaminase (AST) and aspartate transaminase (ALT)).

### Histopathology

After excision from the mice, the liver tissue samples were fixed in 10% formaldehyde at 25°C and then embedded in paraffin. Hematoxylineosin was then used to stain the serial paraffin sections (4 µm) for conventional morphological evaluation under an optical microscope.

### Immunofluorescence and Immunohistochemical Staining

Immunofluorescence and Immunohistochemical (IHC) staining were carried out according to the protocol described previously (Yang et al., 2019).

### Cell Culture

The L-02 cell line, which belong to normal human liver cell, was obtained from Shanghai Institute of Materia Medica, Chinese Academy of Sciences (SIMM) and stored in DMEM with 10% fetal bovine serum and 1% antibiotic-antimitotic reagent. For the treatments, the L-02 cells were plated in 6-well plates at 2 × 10^5^ per well. Twenty-four hours after plating, LPS was added to the cells, and sterile PBS was used as the negative control. puerarin was then added to the cells treated with LPS. Twenty-four hours after treatment, the supernatant and total protein were collected for ELISA and Western blot (WB) assays.

### Transfection Small Interfering RNA

The L-02 cells were plated in a 6-well plate (2 × 10^5^/well) and cultured for 24 h. The transfection of cells was performed according to the method described previously (Zhou et al., 2016; Yang et al., 2019). The siRNA sequences used are listed in Table 1. All experiments were performed in triplicate.

**TABLE 1:** Sequences used in transfection.

Gene	Sequences
ZEB2-siRNA	5′-GAA​GCU​ACG​UAC​UUU​AAU​ATT-3′
	5′-UAU​UAA​AGU​ACG​UAG​CUU​CTT-3′
ZEB2-NC	5′-UUC​UCC​GAA​CGU​GUC​ACG​UTT-3′
	5′-ACG​UGA​CAC​GUU​CGG​AGA​ATT-3′

### Plasmid Construction

The ZEB2 overexpression plasmid was synthesized by amplifying the complementary DNA (cDNA) coding for ZEB2 cDNA and inserting the cDNA coding for ZEB2 into the target vectors using Gateway cloning (Invitrogen, California, United States ). Using the restriction sites XbaI and BamHI, the N-terminal region encoded with ZEB2 containing the predicted CARD domain was cloned into the pEGFP-C2 vector.

ZEB2-F: 5′-GGG​GTA​CCC​CAT​GAA​GCA​GCC​GAT​CAT-3′.
EB2-R: 5′-GCT​CTA​GAG​CTC​ACA​TGC​CAT​CTT​CC-3′.

The manufacturer’s instructions were followed for cell transfection with Lipofectamine™ 2000 (ThermoFisher, California, United States ).

### Western Blot

Sample proteins were transferred to 0.22 μm PVDF membranes (Millipore, Massachusetts, United States ) after separation by 10% PAGE electrophoresis, followed by blocking for 2 h in 5% skimmed milk with tris-buffered saline and Polysorbate 20 (Tween 20) (TBST) buffer. The membranes were then incubated overnight with the primary antibodies at 4°C. The HRP-conjugated secondary antibodies were incubated onto membranes. The protein bands were visualized using a chemiluminescent detection system. β-Actin was used as internal control for the protein. ImageJ computer software (https://imagej.nih.gov/ij/) was used to analyze the density of the immunoreactive bands.

### RNA Extraction and Quantitative Real-Time PCR (qRT-PCR)

qRT-PCR was performed as described previously (Yang et al., 2017; Yang et al., 2019). Total RNA was isolated for cDNA synthesis. The expression levels of the indicated genes were estimated by real-time PCR using SYBR^®^ Green Master (BioRad, United States ). The PCR results for glyceraldehyde 3-phosphate dehydrogenase (GAPDH) were used as internal controls. The primers used for the PCR are listed in Table 2.

**TABLE 2:** Primers used in real-time qRT-PCR.

Gene	Primer sequence
Human	
IL-6	Forward:5′-CACACAGACAGCCACTCACC-3′
	Reverse:5′-AGTGCCTCTTTGCTGCTTTC-3′
TNF-α	Forward:5′-AACCTCCTCTCTGCCATCAA-3′
	Reverse:5′-CTGAGTCGGTCACCCTTCTC-3′
IL-1β	Forward:5′-GGACAAGCTGAGGAAGATGC-3′
	Reverse:5′-TCGTTATCCCATGTGTCG AA-3′
ZEB2	Forward:5′-CACACACATACACAGAAAGGA-3′
	Reverse: 5′-ATA​ACA​GGA​GGC​ATA​GCA​TT-3′
GAPDH	Forward:5′-ACCACAGTCCATGCCATCAC-3′
	Reverse:5′-TCCACCACCCTGTTGCTGTA-3′
Mouse	
IL-6	Forward:5′-CAAAGCCAGAGTCCTTCAGAG-3′
	Reverse:5′-GCCACTCCTTCTGTGACTCC-3′
TNF-α	Forward:5′-CCTCCTCTTTTGCTTATGTT-3′
	Reverse:5′-CAATTACAGTCACGGCTC-3′
IL-1β	Forward:5′-ACGGACCCCAAAAGATGAAG-3′
	Reverse:5′-TTCTCCACAGCCACAATGAG-3′
ZEB2	Forward:5′-GCTACACGTTCGCCTACCG-3′
	Reverse:5′-CCTTGGGTTAGCATTTGGTGC-3′
GAPDH	Forward:5′-AGGTCGGTGTGAACGGATTTG-3′
	Reverse:5′-GTAGACCATGTAGTTGAGGTCA-3′

### Enzyme-Linked Immunosorbent Assay

Human ELISA kits were used to measure the concentrations of serum cytokines according to the manufacturer’s protocols. The levels of IL-1β, IL-6, and tumor necrosis factor-alpha (TNF-α) were detected using the human ELISA kits.

### Statistical Analysis

The data are expressed as the means ± SE of at least three independent experiments and compared using one-way analysis of variance (ANOVA) and the Student Newman–Keuls test using Prism 9.1.0 software (https://www.graphpad.com/scientific-software/prism). *P*-values less than 0.05 were considered statistically significant.

## Results

### Puerarin Protected Mice From ALI Induced by LPS/D-Gal

**FIGURE 1:**
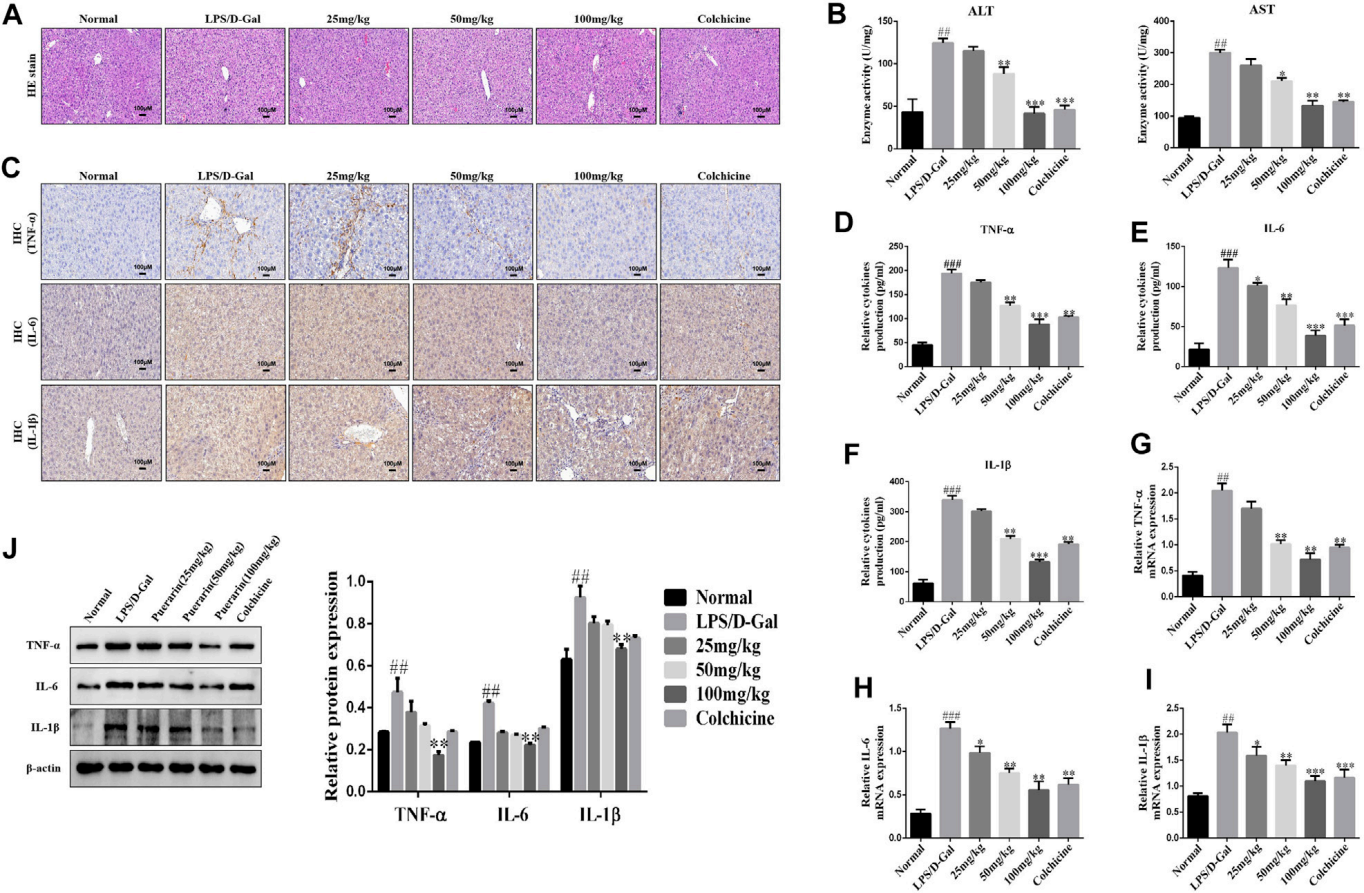
Puerarin protected mice from acute liver injury (ALI) induced by LPS/D-Gal. **(A)**: Hematoxylin and eosin (H and E) staining was conducted, the specimens were examined under a microscope (×100), and inflammatory cell accumulation was counted. **(B)**: Serum activities of ALT and AST were measured (*n* = 6). **(C)**: Representative immunohistochemical stained images of inflammatory cytokines **(D–F)**: The inflammatory cytokines were measured using ELISA **(G–I)**: qRT-PCR analysis of TNF-α, IL-6, and IL-1β mRNA levels in ALI liver tissues. **(J)**: Western blot analysis of TNF-α, IL-6, and IL-1β protein levels in ALI liver tissues. The assays were performed at least three times, with similar results. Data are shown as the means ± SD (*n* = 3) of one representative experiment; *###p* and *##p* < 0.01 versus normal groups; ***p < 0.001 and **p < 0.01 and *p < 0.05 versus the LPS/D-Gal group.

To evaluate the protective role of PUERARIN in ALI in mice, a model of ALI was constructed using LPS/D-Gal, in which the histological changes in ALI were assessed. As demonstrated in Figure 1A, the liver tissues in the control group showed a normal liver structure, while those in the LPS/D-Gal group showed pathological changes, including severe liver structure destruction, cytoplasmic vacuolization, extensive hemorrhaging, and obvious inflammatory cell infiltration. However, treatment with puerarin (25, 50, 100 mg/kg) significantly reduced the pathological process in the LPS/D-Gal-treated mice livers, such that the well-organized hepatic lobular structure and inflammatory cell infiltration were reduced. In addition, the plasma ALT and AST activity levels were also detected, which are two important biochemical indicators of liver failure. The results showed that their levels were remarkably enhanced by administration of LPS/D-Gal (Figure 1B). However, compared with the LPS/D-Gal group, puerarin significantly reduced the activity of ALT and AST (Figure1B).

Next, to further determine the extent of the protective effect of puerarin in LPS/D-Gal-induced ALI, the levels of IL-1β, TNF-α, and IL-6 were monitored. IHC and ELISA analyses showed that, compared with the normal group, LPS/D-Gal increased the IL-1β, TNF-α, and IL-6 expression levels. However, puerarin decreased the IL-1β, TNF-α, and IL-6 expression levels to a remarkable degree compared with the LPS/D-Gal group (Figures 1C–F). Similarly, compared with the LPS/D-Gal group, the protein and mRNA levels of IL-1β, TNF-α, and IL-6 were increased by LPS/D-Gal compared with the normal group, which was inhibited by puerarin (Figures 1G–J). Collectively, these results showed that puerarin had a significant anti-inflammatory activity and a preventative role in LPS/D-Gal-induced acute liver failure.

### Puerarin Reduced the Inflammatory Responses in LPS-Induced L-02 Cells

**FIGURE 2:**
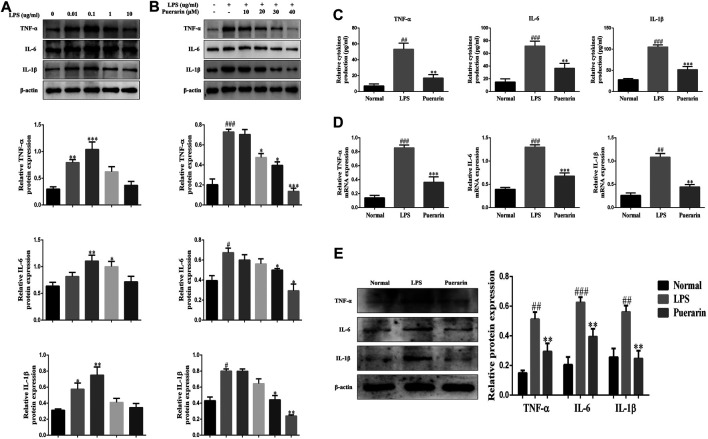
Puerarin reduced the inflammatory responses in LPS-induced L-02 cells. **(A)** and **(B)**: The protein expression levels of TNF-α, IL-6, and IL-1β were measured by Western blot (WB) analyses. **(C)**: The expression levels of TNF-α, IL-6, and IL-1β were measured by ELISA. **(D)**: The mRNA levels of TNF-α, IL-6, and IL-1β were measured using qRT-PCR. **(E)**: The protein expression levels of TNF-α, IL-6, and IL-1β were measured using WB analyses. The assays were performed at least three times, with similar results. Data are shown as the means ± SD (*n* = 3) of one representative experiment; *###p* < 0.001 and *##p* < 0.01, *#p* < 0.05 versus normal groups; ***p < 0.001and **p < 0.01 and *p < 0.05 versus the LPS group.

In order to evaluate the effect of puerarin on inflammation in ALI, we established an ALI model in L-02 cells using LPS (0.01, 0.1, 1, 10 μg/ml, 24 h). The Western Blot results showed that LPS significantly increased IL-1β, TNF-α, and IL-6 expression levels compared with the normal group, and L-02 cells were treated with LPS to reach a peak value of 0.1 μg/ml (24 h) (Figure 2A). Moreover, LPS (0.1 μg/ml, 24 h) and puerarin (10, 20, 30, 40 μM, 24 h) were used to stimulating the L-02 cells. The above results showed that the protein levels of IL-1β, TNF-α, and IL-6 were reduced in the LPS-induced L-02 cells effectively, which peaked with the puerarin treatment at 40 μM (Figure 2B). Additionally, the results of the ELISA showed that the levels of IL-1β, TNF-α, and IL-6 were enhanced by LPS and reduced by puerarin (Figure 2C). The qRT-PCR and WB results showed that the protein and mRNA levels of IL-1β, TNF-α, and IL-6 were inhibited in the L-02 cells induced by LPS (0.1 μg/ml, 24 h) and puerarin (40 μM, 24 h) (Figures 2D,E). Collectively, these results suggested that higher doses of puerarin had an obvious anti-inflammatory effect in the LPS-mediated L-02 cells.

### Puerarin Elevated the Expression Level of ZEB2 in ALI and LPS-Induced L-02 Cells in Mice

**FIGURE 3:**
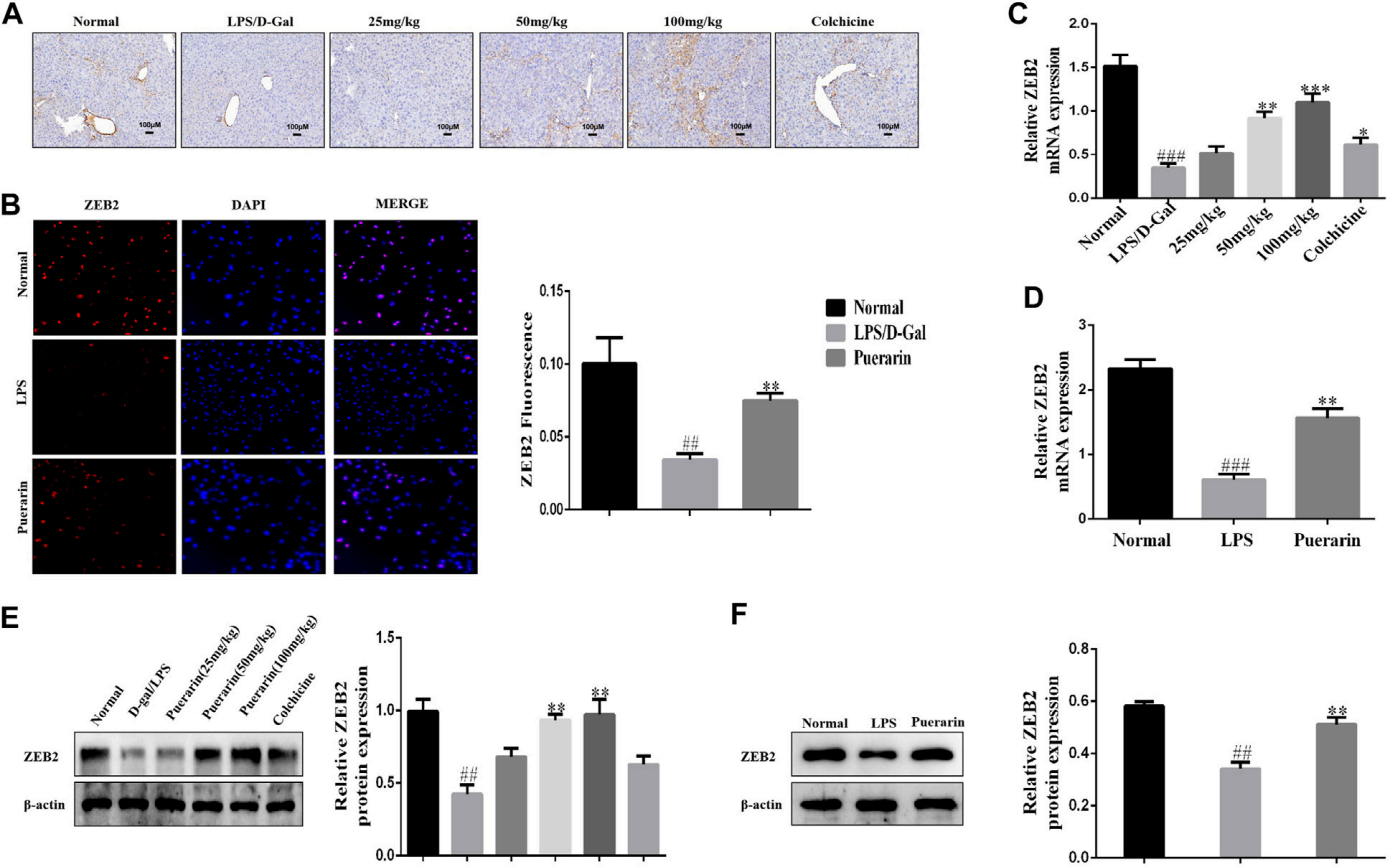
Puerarin elevated the expression level of ZEB2 with ALI and LPS-induced L-02 cells in mice. **(A)**: Representative immunohistochemical stained images of ZEB2. **(B)**: Representative immunofluorescence stained images of ZEB2. **(C)** and **(D)**: The mRNA expression levels of ZEB2 were measured using qRT-PCR in liver tissues with ALI and LPS-induced L-02 cells. **(E)** and **(F)**: The protein expression levels of ZEB2 were measured using WB analyses in liver tissues with ALI and LPS-induced L-02 cells. The assays were performed at least three times, with similar results. Data are shown as the means ± SD (*n* = 3) of one representative experiment; *###p* < 0.001 and *##p* < 0.01 versus normal groups; ***p < 0.001 and **p < 0.01 and *p < 0.05 versus the LPS group.

Growing evidence has shown that ZEB2 is closely associated with inflammatory cytokine secretion in inflammation-related diseases (Kajita et al., 1980; Omilusik et al., 2015; Yang et al., 2021). Thus, we detected the expression level of ZEB2 in LPS/D-Gal-induced mice and LPS-induced L-02 cells using qRT-PCR. As presented at Figure 3A, the ZEB2 expression level was significantly attenuated by LPS/D-Gal *in vivo* in comparison with the normal group. However, compared with the LPS/D-Gal group, puerarin promoted the expression of ZEB2 significantly. These results were further confirmed by WB and qRT-PCR (Figures 3C,E). The ZEB2 expression level was then measured in the LPS-induced L-02 cells. The results of IF, WB, and qRT-PCR showed that LPS suppressed the protein and mRNA levels of ZEB2 compared with the normal group in the L-02 cells. However, puerarin dramatically impaired the downregulation of ZEB2 (Figures 3B,D,F). These results showed that puerarin promoted the ZEB2 expression level in the LPS-induced L-02 cells.

### Lipidosome-Mediated Transduction Leading to Overexpression or Knockdown of ZEB2 in L-02 Cells

**FIGURE 4:**
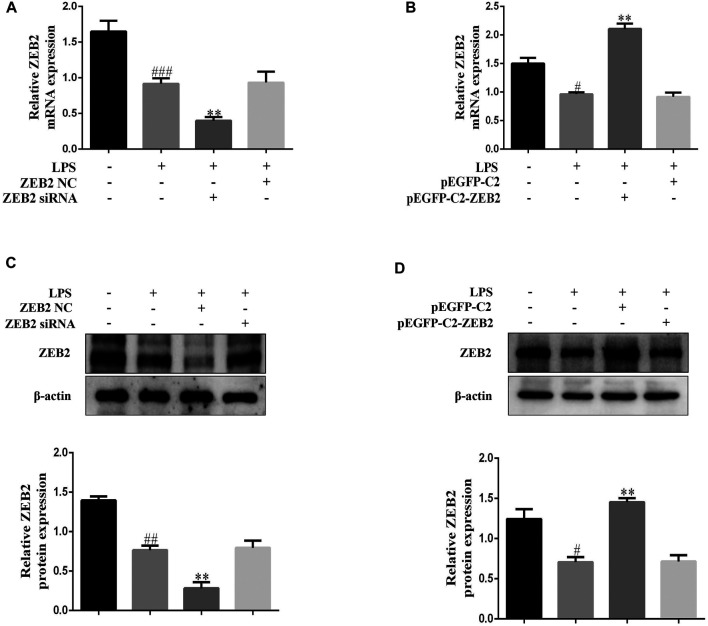
Lipidosome-mediated transduction leading to overexpression or knockdown of ZEB2 in L-02 cells. **(A)** and **(B)**: The mRNA expression levels of ZEB2 were measured using qRT-PCR in LPS-induced L-02 cells transfected with ZEB2 siRNA and pEGFP-C2-ZEB2 **(C)** and **(D)**: The protein expression levels of ZEB2 were measured using WB analyses in LPS-induced L-02 cells transfected with ZEB2 siRNA and pEGFP-C2-ZEB2. The assays were performed at least three times, with similar results. Data are shown as the means ± SD (*n* = 3) of one representative experiment; *###p* < 0.001 and *##p* < 0.01, *#p* < 0.05 versus normal groups; **p < 0.01 and *p < 0.05 versus the LPS group.

To investigate the ZEB2 effect on ALI, we inhibited and promoted the expression of ZEB2 using targeted siRNA and pEGFP-C2-ZEB2 in L-02 cells, respectively. The ZEB2 siRNA and the pEGFP-C2-ZEB2 were transfected into L-02 cells using lipidosomes. The above results showed that ZEB2-siRNA stimulated a clear reduction in ZEB2 protein and mRNA expression levels in L-02 cells in comparison with the ZEB2-NC group (Figures 4A,C). In contrast, the protein and mRNA levels of ZEB2 were elevated by pEGFP-C2-ZEB2 (Figures 4B,D). The above data offer a strategy for studying the downstream signaling pathway of ZEB2 in L-02 cells.

### ZEB2 Inhibited the Expression of IL-6, TNF-α, and IL-1β in LPS-Induced L-02 Cells

**FIGURE 5:**
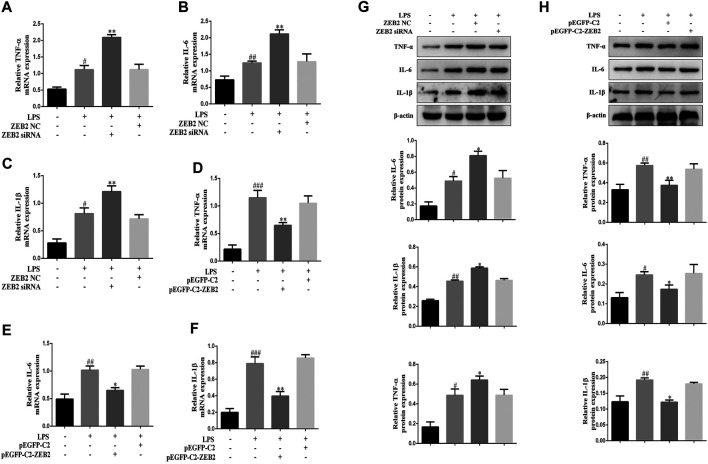
ZEB2 inhibited the expression levels of TNF-α, IL-1β, and IL-6 in LPS-induced L-02 cells. **(A–F)**: The mRNA expression levels of TNF-α, IL-6, and IL-1β were measured using qRT-PCR in L-02 cells transfected with ZEB2 siRNA and pEGFP-C2-ZEB2. **(G)** and **(H)**: The protein expression levels of TNF-α, IL-6, and IL-1β were measured using WB analyses in L-02 cells transfected with ZEB2 siRNA and pEGFP-C2-ZEB2. The assays were performed at least three times, with similar results. Data are shown as the means ± SD (*n* = 3) of one representative experiment; *###p* < 0.001 and *##p* < 0.01, *#p* < 0.05 versus normal groups; **p < 0.01 and *p < 0.05 versus the LPS group.

**FIGURE 6:**
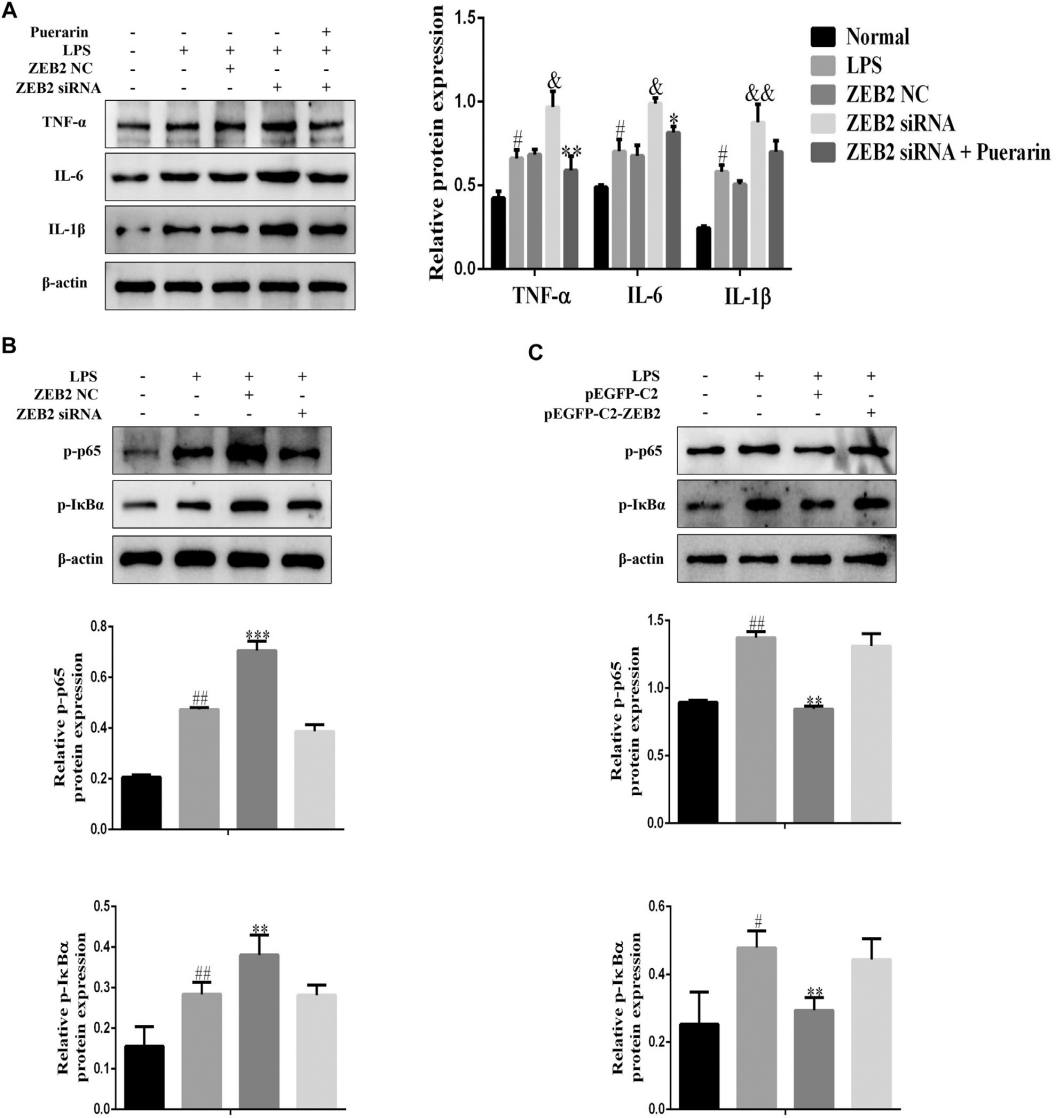
ZEB2 inhibited the LPS-induced activation of the NF-κB signaling pathway in L-02 cells. **(A)**: The expression levels of TNF-α, IL-6, and IL-1β were detected using WB analyses in LPS-induced L-02 cells treated with PUERARIN and transfected with ZEB2 siRNA and pEGFP-C2-ZEB2. **(B)** and **(C)**: The protein expression levels of p-p65 and p-IκBα were detected using WB analyses in LPS-induced L-02 cells transfected with ZEB2 siRNA and pEGFP-C2-ZEB2. The assays were performed at least three times, with similar results. Data are shown as the means ± SD (*n* = 3) of one representative experiment; *###p* < 0.001 and *##p* < 0.01, *#p* < 0.05 versus normal groups; **p < 0.01 and *p < 0.05 versus the LPS group.

To investigate the potential anti-inflammatory effect of ZEB2 in ALI, the levels of pro-inflammatory cytokines, including IL-1β, IL-6, and TNF-α, were monitored in the LPS-induced L-02 cells. The results of qRT-PCR analysis demonstrated that ZEB2-siRNA promoted the levels of IL-6, IL-1β, and TNF-α mRNA compared with the ZEB2-NC group (Figures 5A–C). In contrast, the expression levels of IL-1β, IL-6, and TNF-α were inhibited by pEGFP-C2-ZEB2 (Figures 5D–F). WB analysis also confirmed the inhibiting effect of ZEB2 on LPS-induced IL-6, TNF-α, and IL-1β upregulation at the protein level (Figures 5G,H). Importantly, the expression of TNF-α, IL-1β, and IL-6 was inhibited in L-02 cells co-treated with ZEB2-siRNA and puerarin (Figure 6A). Generally, the results suggested that ZEB2 might act as an important mediator of inflammatory responses in LPS-induced L-02 cells.

### ZEB2 Inhibited LPS-Induced the Activation of NF-κB Signaling Pathway in L-02 Cells

It is clear from the results of several studies that NF-κB signaling is closely related to prototypical pro-inflammatory cytokines that play important roles in the pathogenesis of inflammatory diseases (Lawrence, 2009). Therefore, in order to further explore the anti-inflammatory mechanism of ZEB2 in L-02 cells, WB was used to detect p-p65 and p-IκBα expression in L-02 cells following pEGFP-C2-ZEB2 and ZEB2-siRNA treatment. As shown in Figure 6B, the expression levels of p-p65 and p-IκBα were promoted by LPS. However, pEGFP-C2-ZEB2 significantly repressed the p-p65 and p-IκBα expression levels in L-02 cells. By contrast, knockdown of ZEB2 promoted the expression levels of p-p65 and p-IκBα in LPS-induced L-02 cells (Figure 6C). Collectively, the results showed that the mechanism of ZEB2-inhibited ALI might be associated with activation of the NF-κB signaling pathway.

## Discussion

**FIGURE 7:**
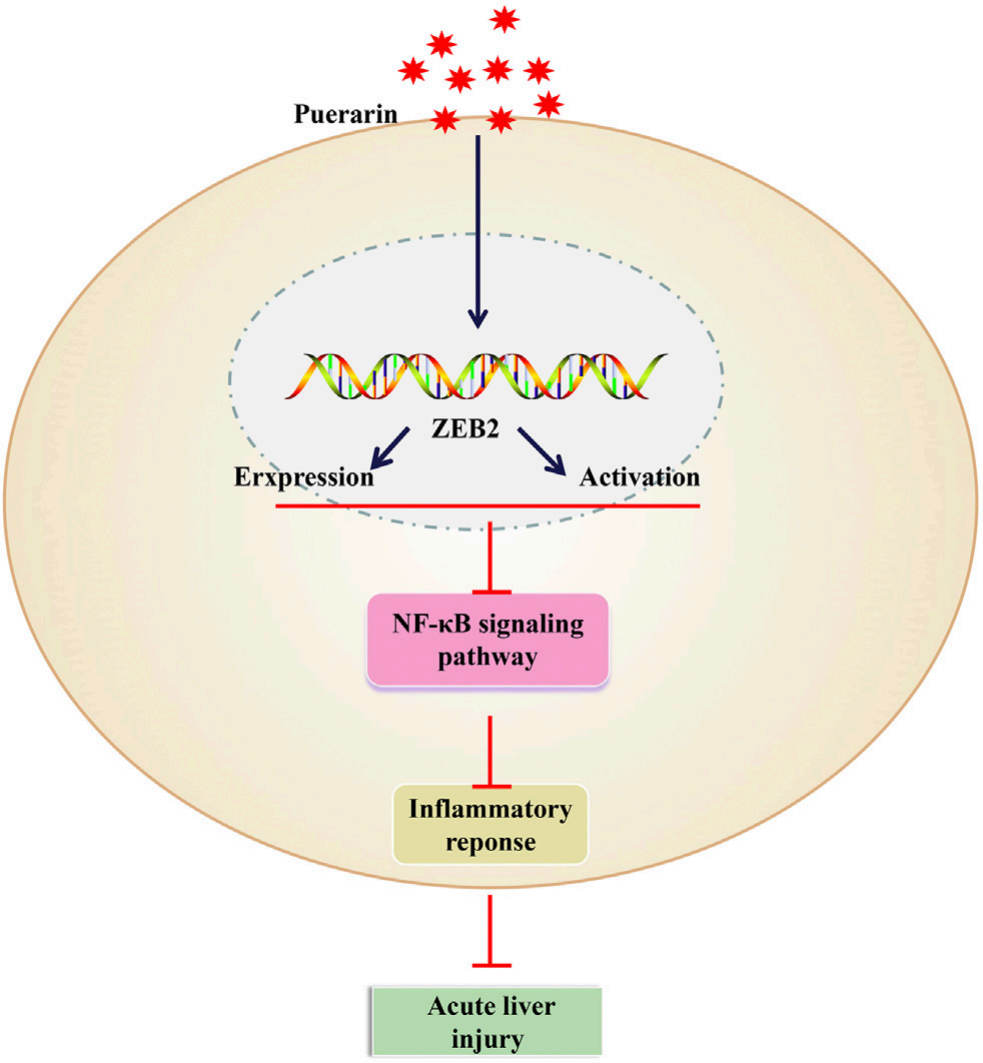
Puerarin upregulated the expression levels of ZEB2 in a dose-dependent manner in LPS-induced L-02 cells. It was then able to inhibit the production of TNF-α, IL-6, and IL-1β by suppressing the activation of the NF-κB signaling pathway.

For all we know, this is first study to investigate the regulatory role and potential molecular mechanisms of puerarin in the development of ALI. Through the results, we confirmed that the expression of IL-1β, TNF-α, and IL-6 could be inhibit by puerarin, thereby attenuating the LPS-induced inflammatory response of L-02 cells and LPS/D-Gal-induced ALI. Furthermore, the expression of ZEB2 was downregulated in ALI, while it was upregulated by puerarin. After that, we also testified that the activation of NF-κB signaling pathway was inhibited by ZEB2. These discoveries may help us to go a step further to understand the molecular role of puerarin and have more opportunities to find novel therapeutic targets for ALI (Figure 7).

ALI is a serious hepatic inflammatory disease with high mortality and morbidity, caused by alcohol abuse, drug side effects, hepatitis viruses, metabolic syndromes, hepatotoxins originating from sepsis, and bacterial infections (Peng et al., 2019). Growing evidence has demonstrated that inflammatory cytokines play a key role in ALI development (Bhatia and Moochhala, 2004). IL-1β can cause cell damage, and the production of IL-1β can lead to lung epithelial injury (Kolb et al., 2001). TNF-α, the earliest endogenous mediator, can enhance the inflammatory response (Gouwy et al., 2005). In addition. IL-6, the main pro-inflammatory cytokine, is pleiotropic and can induce lung edema (Kubo et al., 1998). Therefore, inhibiting the above inflammatory cytokines, which can lead to serious tissue damage, can attenuate ALI induced by LPS (Goodman et al., 2003). Although the etiology varies widely, these conditions induce the activation of immune mechanisms, exacerbate inflammation following the first attack, and might even lead to fatal loss of liver function characterized by excessive hepatocyte death (Kim and Lee, 2013). Currently, the available treatments for ALI/acute liver failure are mainly supportive, protecting hepatocytes and preventing complications caused by severe liver failure, while related treatments for the specific pathogenesis of ALI have not been found (Stravitz and Lee, 2019). In summary, the need to determine related specific targets and targeted drugs/chemotherapies for the treatment of ALI is urgent.

Puerarin is a flavonoid component isolated from Pueraria lobata widely known as Gegen in traditional Chinese medicine and has been clinically used for its protection against inflammation, oxidative stress, and mitochondrial dysfunction (Wang et al., 2016; Zeng et al., 2018; Xiao et al., 2020). Previous research has found that puerarin plays a significantly regulator role in various diseases, including liver damage (Peng et al., 2013). In addition, it has been reported that puerarin have anti-tumor (Kapoor, 2013), anti-inflammatory (Singh et al., 2013) and antioxidant effect (Wang et al., 2014). Interestingly, a finding suggested that puerarin could inhibited LPS-induced acute lung injury through inhibiting inflammatory response (Wang X. et al., 2018). More importantly, Li et al. showed that puerarin could prevent the LPS/D-Gal-induced liver injury in mice, and its mechanisms might be associated with the increments of autophagy and suppression of apoptosis (Li et al., 2018). In the present study, we found that puerarin possesses immunoregulatory and anti-inflammatory effects. Although these studies have shown that puerarin can play an important role in inflammation, the functional role of puerarin in the inflammatory response of ALI remains unknown. Our studies so far have found that LPS stimulated IL-1β, TNF-α, and IL-6 expression in L-02 cells and mice liver tissues with ALI. However, the upregulated expression levels of these cytokines were suppressed by puerarin. These results showed that puerarin can inhibit the inflammatory response so as to resist ALI induced by LPS.

ZEB2, also known as ZFHX1B and SMAD-interacting protein-1 (SIP1), is a DNA-binding transcription regulator. It is a dimerization in the E-box motif in diverse promoters (E-cadherin promoter) and downregulates E-cadherin and other epithelial genes (Verschueren et al., 1999). Numerous studies have revealed that ZEB2 induces the epithelial-to-mesenchymal transition (EMT) efficiently (Fardi et al., 2019). Additionally, a previous study has shown that miR-498 targets ZEB2 to inhibit invasion, migration, and proliferation in liver cancer cells (Zhang et al., 2019), suggesting that the level of ZEB2 is abnormal in some diseases, including liver disease. Importantly, Ding et al. (2018) demonstrated that LPS promotes inflammatory cytokine secretion. However, ZEB2 inhibited inflammatory cytokine secretion in acute kidney injury and was reported to be related to the NF-κB signaling pathway (Ding et al., 2018). Interestingly, our group’s previous study showed that paeonol derivative-6 activates ZEB2 to attenuate inflammation in ALI (Yang et al., 2021). In this study, our results have demonstrated that LPS reduces the production of inflammatory cytokines in L-02 cells and ALI liver tissues. However, the inflammatory response was inhibited by puerarin. Moreover, puerarin at a higher dose (100 mg/kg) had a better anti-inflammatory effect than it had in the lower dose (25 mg/kg) group. Furthermore, ZEB2 expression was found to be increased by puerarin in LPS-induced L-02 cells and liver tissues with ALI. More importantly, knockdown of ZEB2 elevated the IL-1β, TNF-α, and IL-6 expression levels in LPS-induced L-02 cells compared with ZEB2-NC. In contrast, compared with the pEGFP-C2 group, pEGFP-C2-ZEB2 induced a significant inhibition of the IL-1β, TNF-α, and IL-6 expression levels in LPS-induced L-02 cells. Furthermore, the results showed that the anti-inflammatory effects of puerarin were inhibited after ZEB2 had been blocked. These results showed that puerarin inhibited the inflammatory responses through ZEB2. Moreover, puerarin was also considered to activate ZEB2 and show anti-inflammatory effects.

Immune responses and inflammation are known to be regulated following NF-κB activation (Vallabhapurapu and Karin, 2009). Generally, NF-κB is localized in the cytoplasm through a family of inhibitory proteins, the NF-κB (IκBs). Following activation by LPS, the NF-κB unit p65 separates from IκB and transfers to the nucleus and regulates the expression of inflammatory cytokines (Lawrence et al., 2005). Studies have shown that lung injury in a mouse model is reduced following inhibition of the activation of NF-κB (Everhart et al., 2006). Multiple strategies and agents have been shown to inhibit the activation of NF-κB and reduce the recruitment of neutrophils into lung tissue (Shen et al., 2009; Yunhe et al., 2012). Significantly, inhibiting the NF-κB signaling pathway can reduce inflammation effectively and prevent joint destruction in experimental models of arthritis, as the pathway plays an effective role in regulating a variety of inflammatory reactions (Li et al., 2015; Zhang et al., 2017). In this study, our results showed that pEGFP-C2-ZEB2 induces a significant inhibition of p-p65 and p-IκBα expression levels in LPS-induced L-02 cells, in comparison with pEGFP-C2. However, knockdown of ZEB2 significantly increased p-p65 and p-IκBα expression levels in LPS-induced L-02 cells compared with the ZEB2-NC group. Collectively, these results show that ZEB2 subsequently inhibited activation of the NF-κB pathway in LPS-induced L-02 cells.

In summary, our results have demonstrated that LPS/D-Gal induced the production of inflammatory cytokines in ALI liver tissues, while puerarin inhibited their production in ALI. Furthermore, the ZEB2 expression level was reduced in ALI. The upregulated expression level of ZEB2 blocks ALI inflammatory responses by means of the NF-κB signaling pathway. Therefore, the results of this study show that puerarin might serve as an activator of ZEB2 via NF-κB signaling, thereby inhibiting inflammation. Our work has confirmed the effects of puerarin on the production of inflammatory cytokines *in vitro*. Furthermore, the potential application of puerarin to act as a novel lead compound in ALI anti-inflammatory therapy has also been highlighted.

## Data Availability

The raw data supporting the conclusions of this article will be made available by the authors, without undue reservation.
