# Supplementary figures and images for: Puerarin Prevents Acute Liver Injury via Inhibiting Inflammatory Responses and ZEB2 Expression
Source: Front Pharmacol. 2021 Aug 6;12:727916. doi: 10.3389/fphar.2021.727916 (PMC8378253; doi:10.3389/fphar.2021.727916)

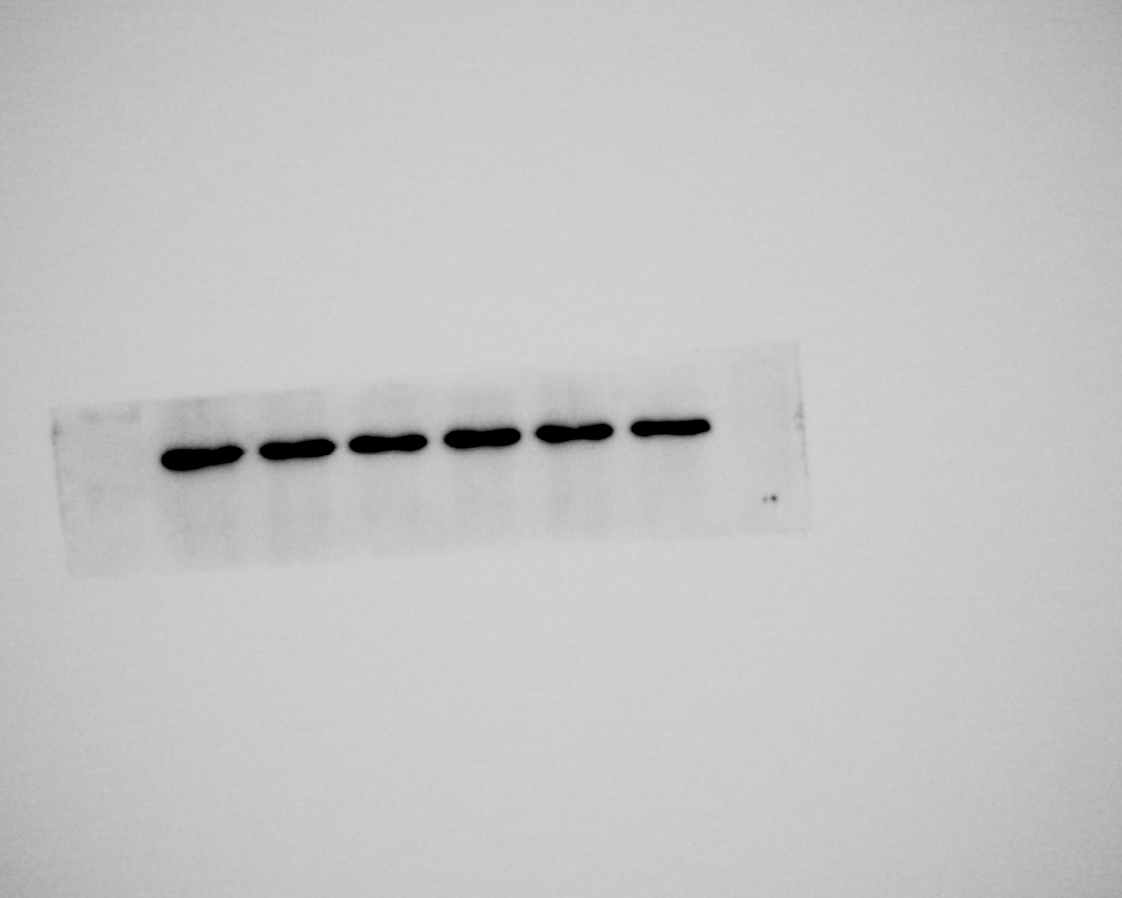

Supplement: Supplementary file 1 [file DataSheet1.ZIP › Orginal Images of Western Blot/Figure 1/1.jpg]

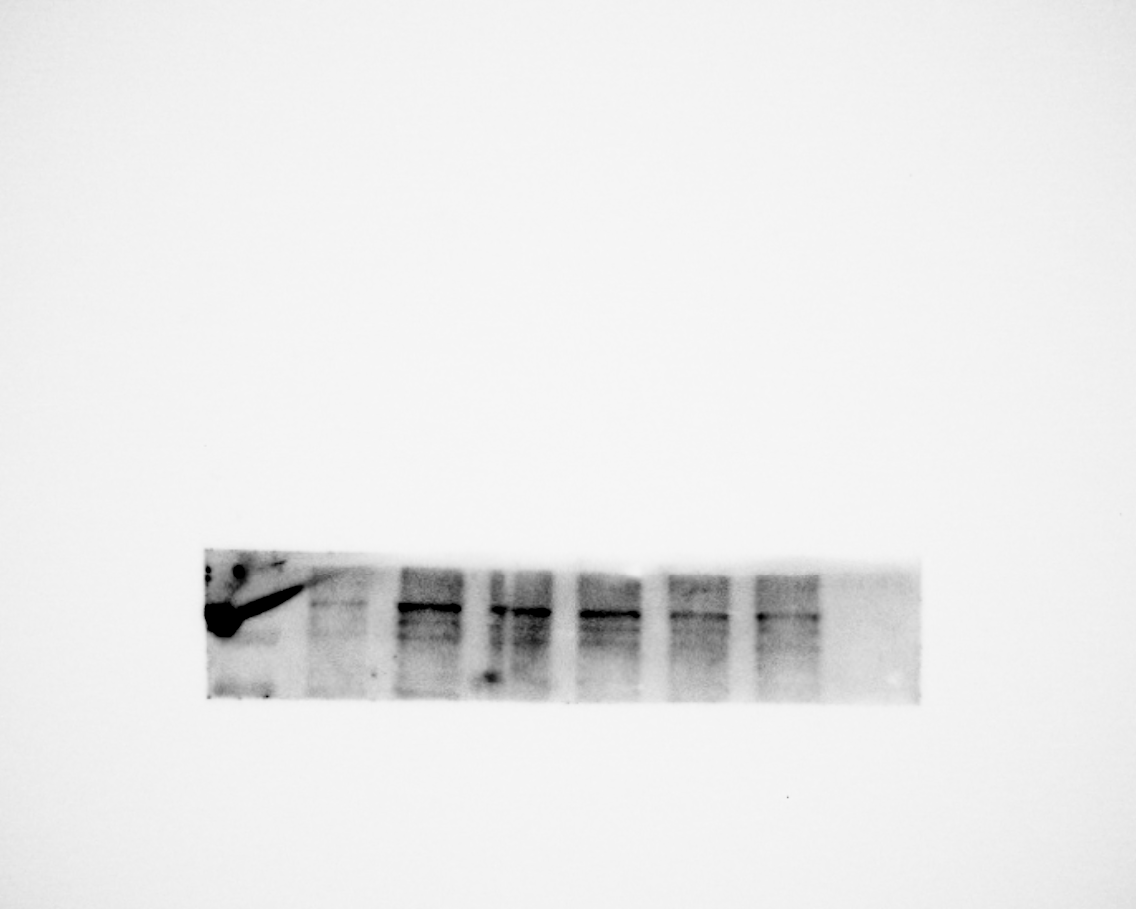

Supplement: Supplementary file 1 [file DataSheet1.ZIP › Orginal Images of Western Blot/Figure 1/2.tif]

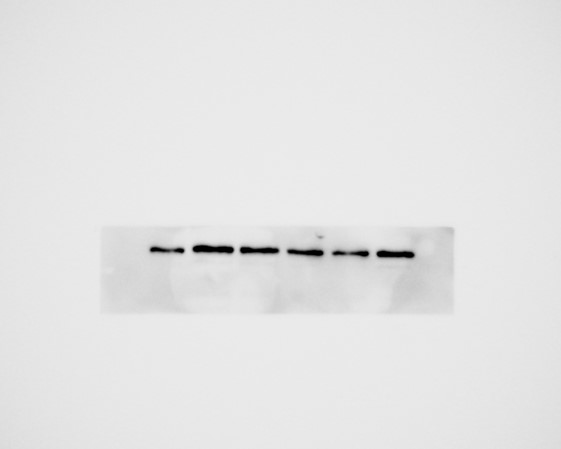

Supplement: Supplementary file 1 [file DataSheet1.ZIP › Orginal Images of Western Blot/Figure 1/3.jpg]

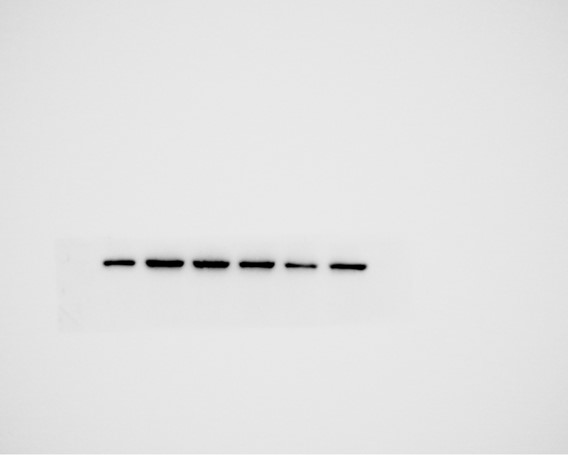

Supplement: Supplementary file 1 [file DataSheet1.ZIP › Orginal Images of Western Blot/Figure 1/4.jpg]

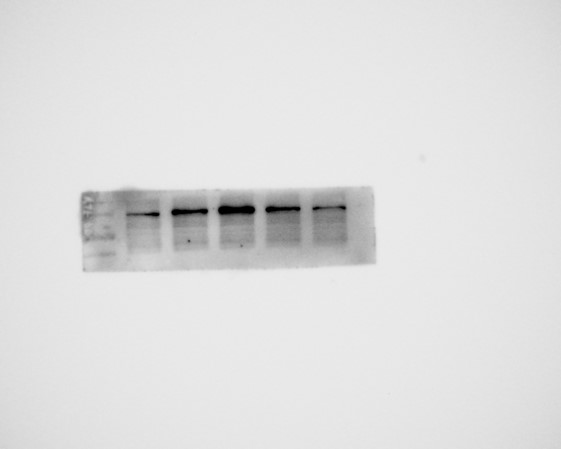

Supplement: Supplementary file 1 [file DataSheet1.ZIP › Orginal Images of Western Blot/Figure 2/1.jpg]

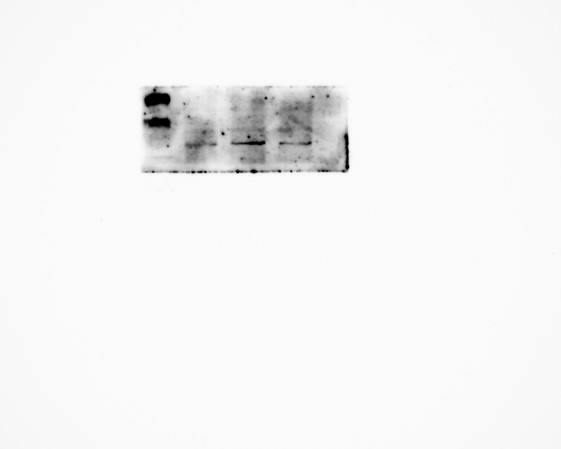

Supplement: Supplementary file 1 [file DataSheet1.ZIP › Orginal Images of Western Blot/Figure 2/10.jpg]

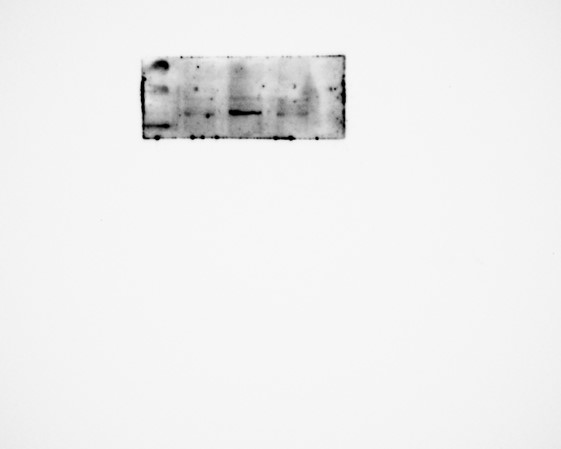

Supplement: Supplementary file 1 [file DataSheet1.ZIP › Orginal Images of Western Blot/Figure 2/11.jpg]

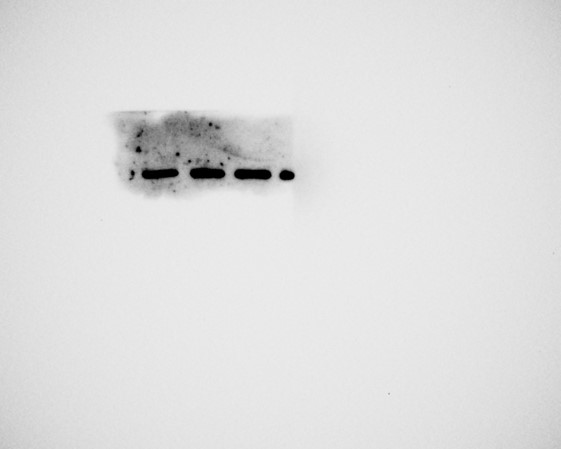

Supplement: Supplementary file 1 [file DataSheet1.ZIP › Orginal Images of Western Blot/Figure 2/12.jpg]

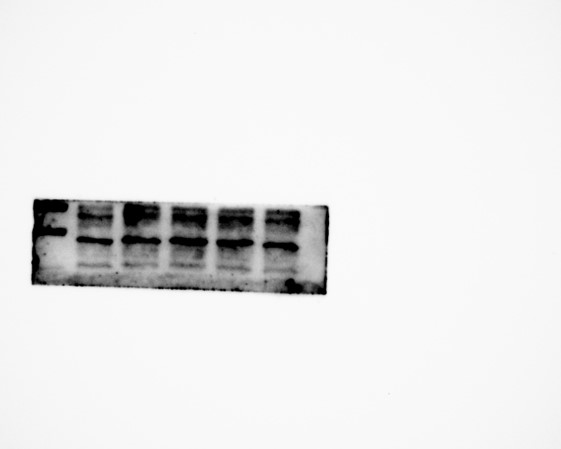

Supplement: Supplementary file 1 [file DataSheet1.ZIP › Orginal Images of Western Blot/Figure 2/2.jpg]

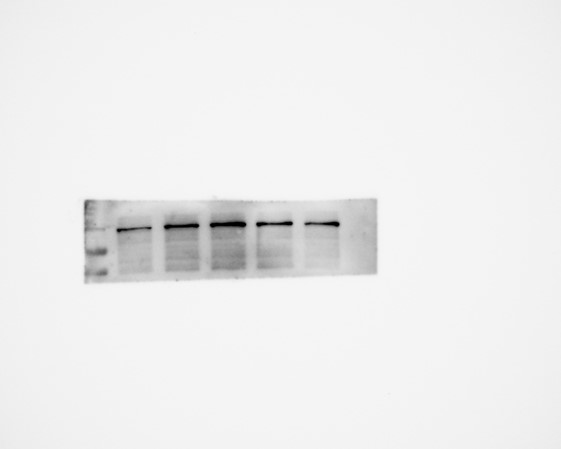

Supplement: Supplementary file 1 [file DataSheet1.ZIP › Orginal Images of Western Blot/Figure 2/3.jpg]

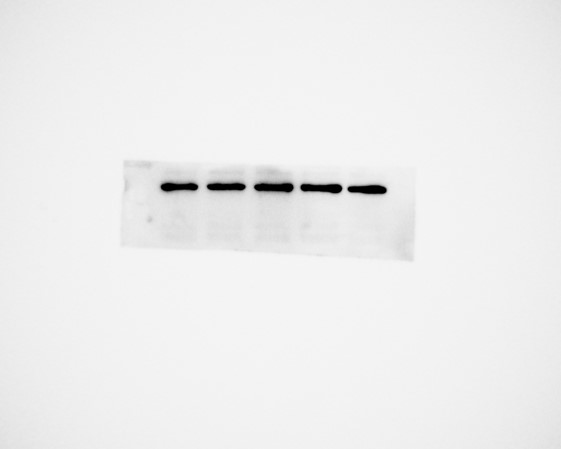

Supplement: Supplementary file 1 [file DataSheet1.ZIP › Orginal Images of Western Blot/Figure 2/4.jpg]

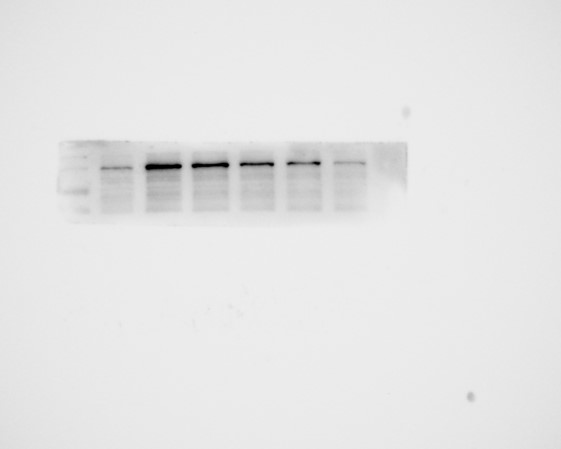

Supplement: Supplementary file 1 [file DataSheet1.ZIP › Orginal Images of Western Blot/Figure 2/5.jpg]

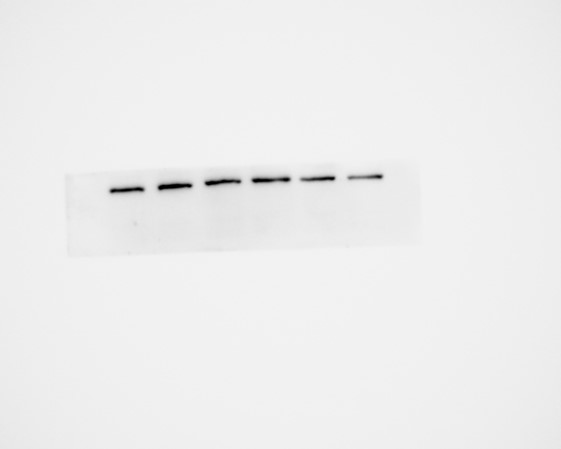

Supplement: Supplementary file 1 [file DataSheet1.ZIP › Orginal Images of Western Blot/Figure 2/6.jpg]

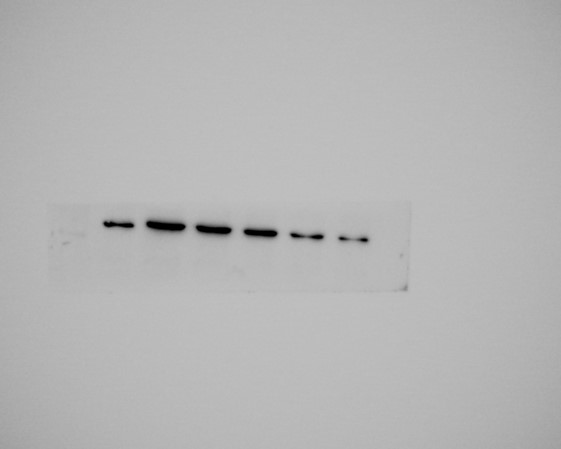

Supplement: Supplementary file 1 [file DataSheet1.ZIP › Orginal Images of Western Blot/Figure 2/7.jpg]

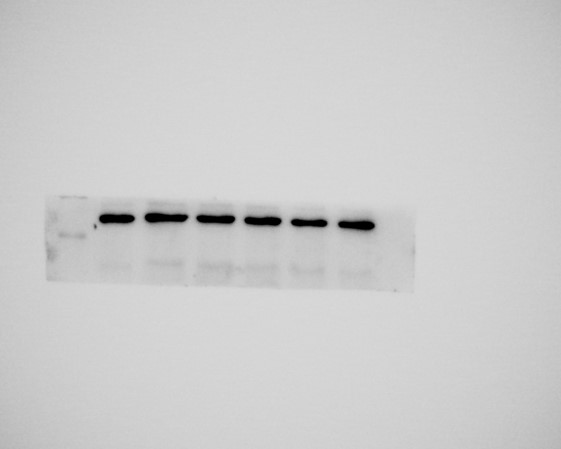

Supplement: Supplementary file 1 [file DataSheet1.ZIP › Orginal Images of Western Blot/Figure 2/8.jpg]

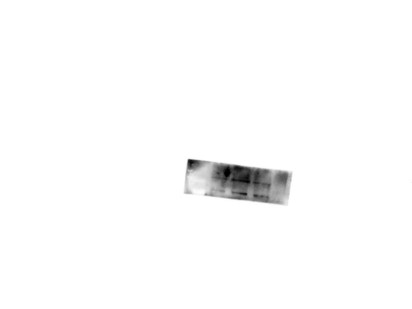

Supplement: Supplementary file 1 [file DataSheet1.ZIP › Orginal Images of Western Blot/Figure 2/9.jpg]

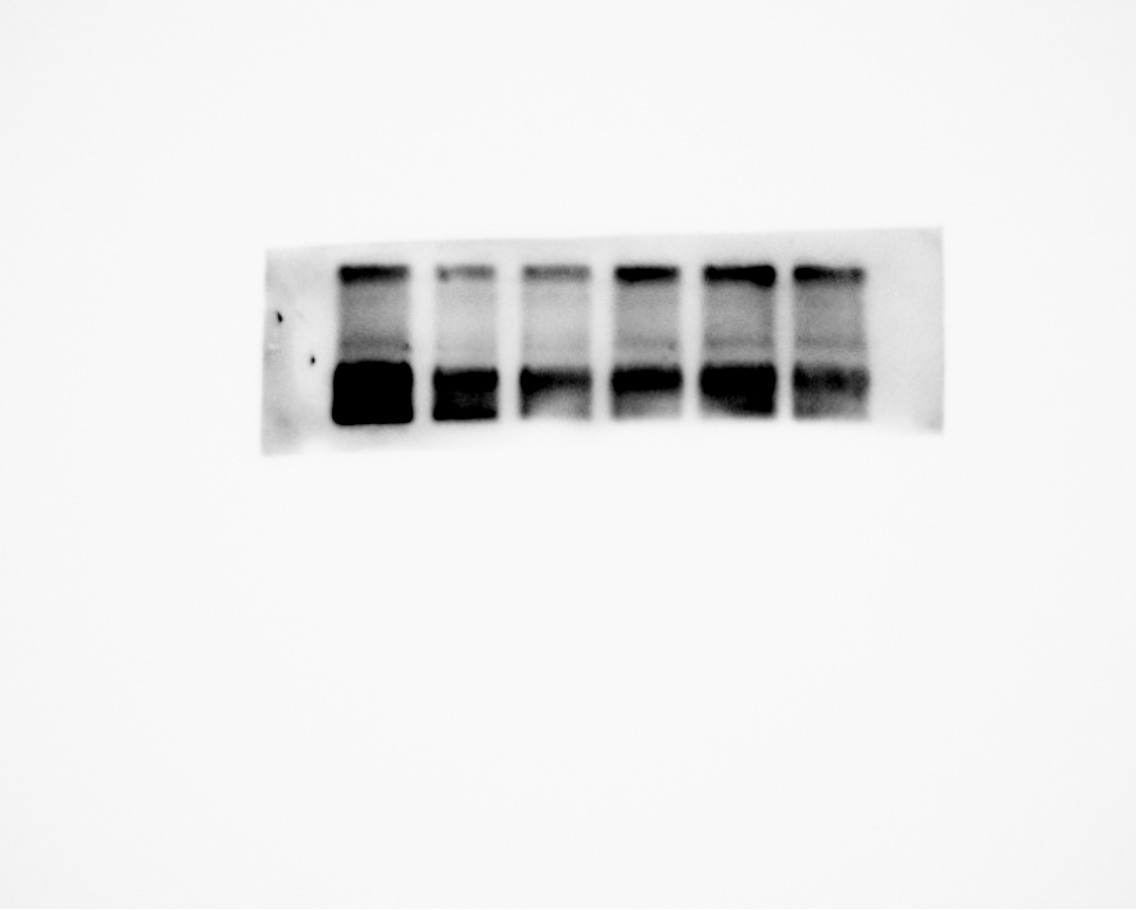

Supplement: Supplementary file 1 [file DataSheet1.ZIP › Orginal Images of Western Blot/Figure 3/1.jpg]

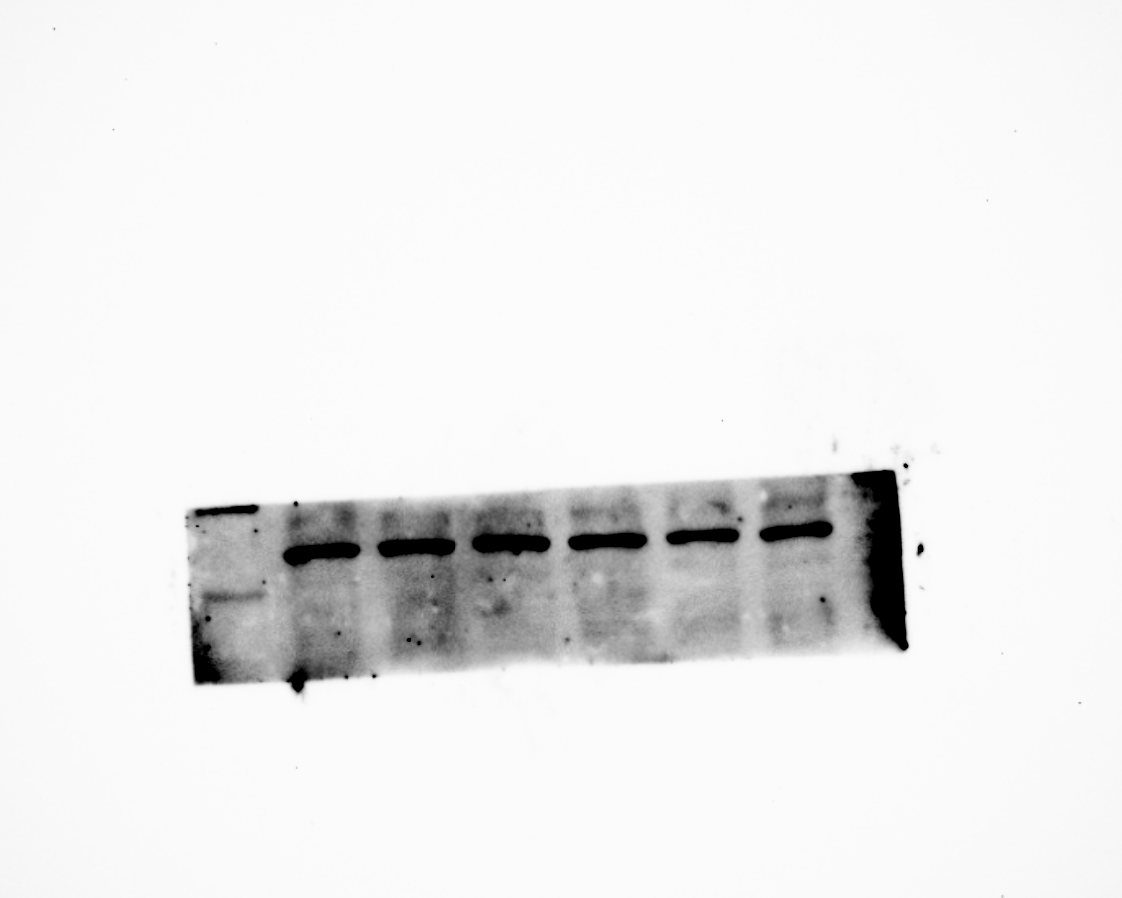

Supplement: Supplementary file 1 [file DataSheet1.ZIP › Orginal Images of Western Blot/Figure 3/2.tif]

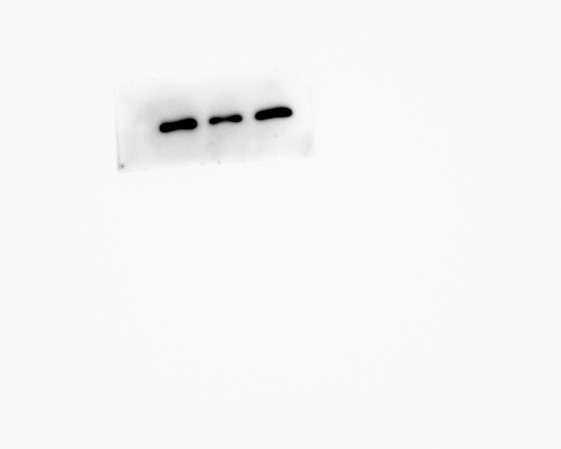

Supplement: Supplementary file 1 [file DataSheet1.ZIP › Orginal Images of Western Blot/Figure 3/3.jpg]

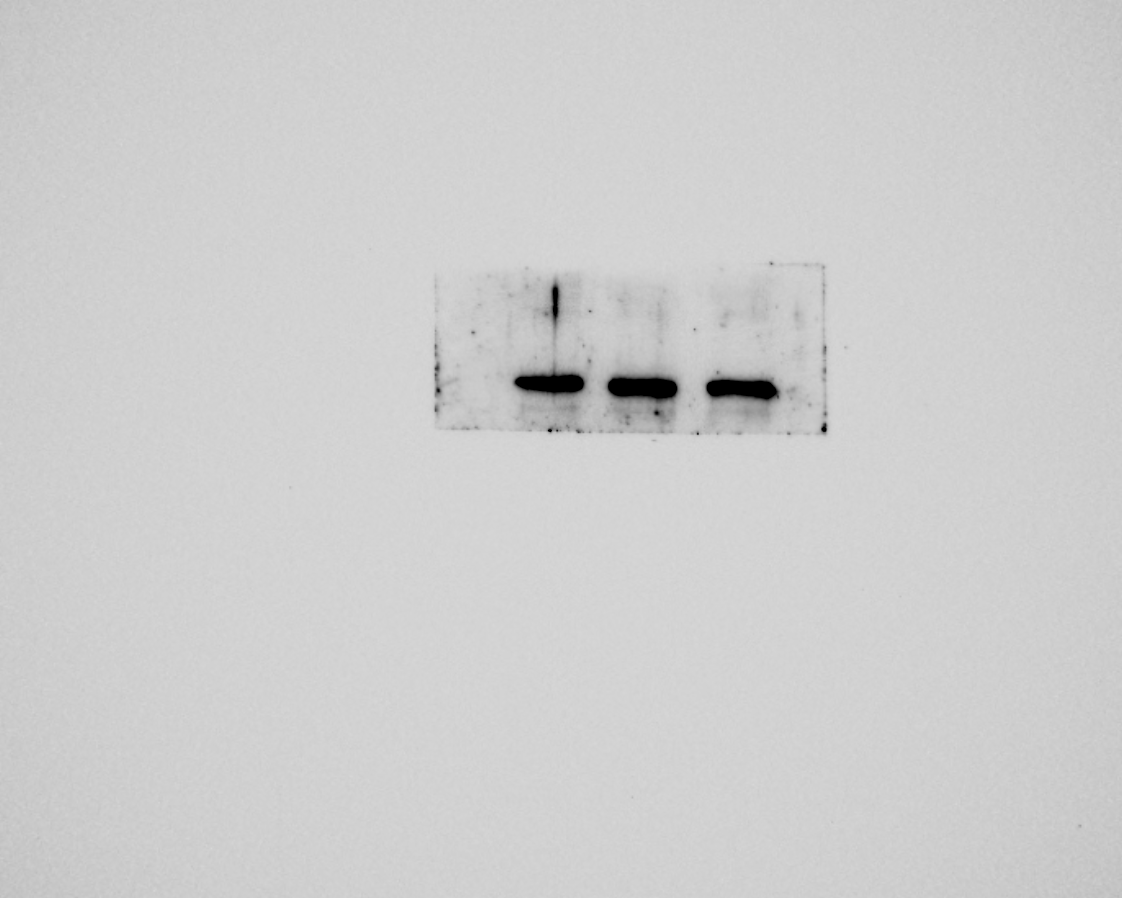

Supplement: Supplementary file 1 [file DataSheet1.ZIP › Orginal Images of Western Blot/Figure 3/4.tif]

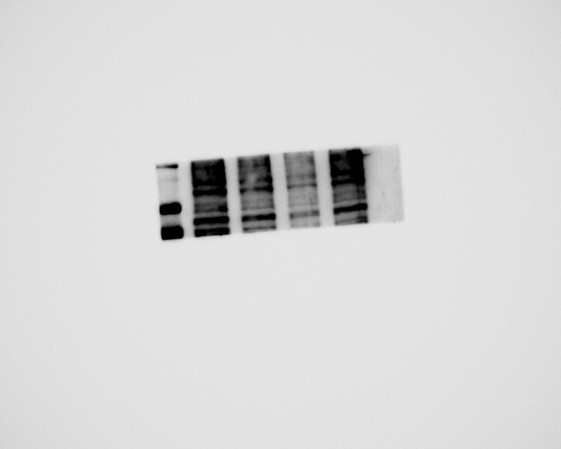

Supplement: Supplementary file 1 [file DataSheet1.ZIP › Orginal Images of Western Blot/Figure 4/1.jpg]

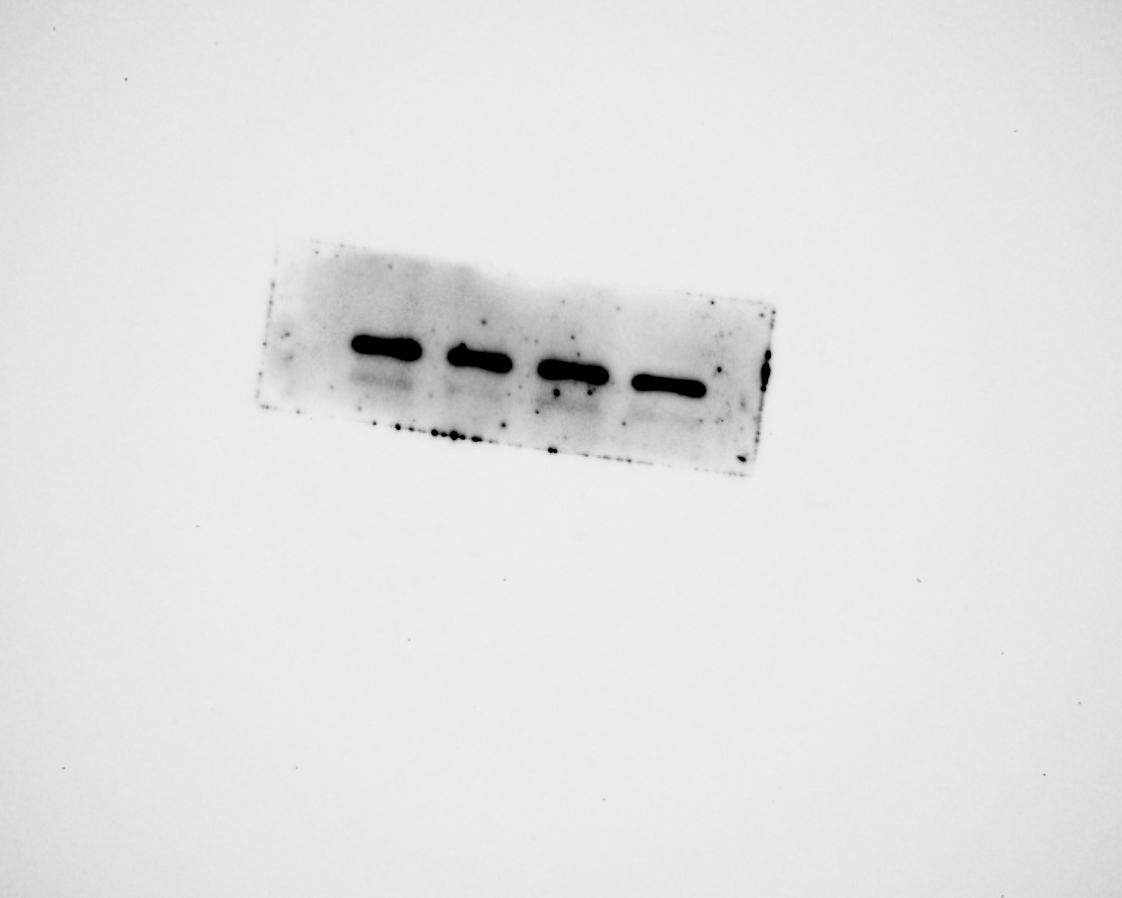

Supplement: Supplementary file 1 [file DataSheet1.ZIP › Orginal Images of Western Blot/Figure 4/2.jpg]

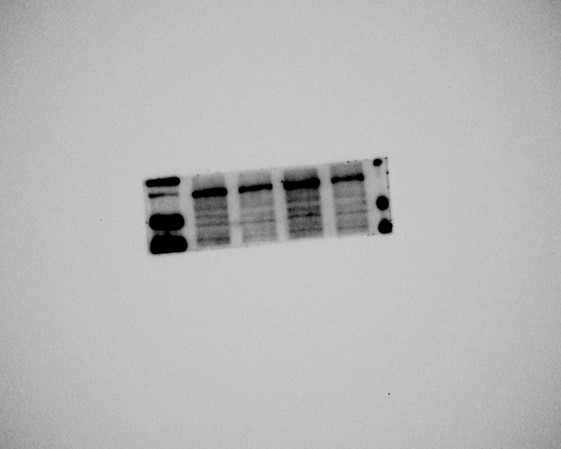

Supplement: Supplementary file 1 [file DataSheet1.ZIP › Orginal Images of Western Blot/Figure 4/3.jpg]

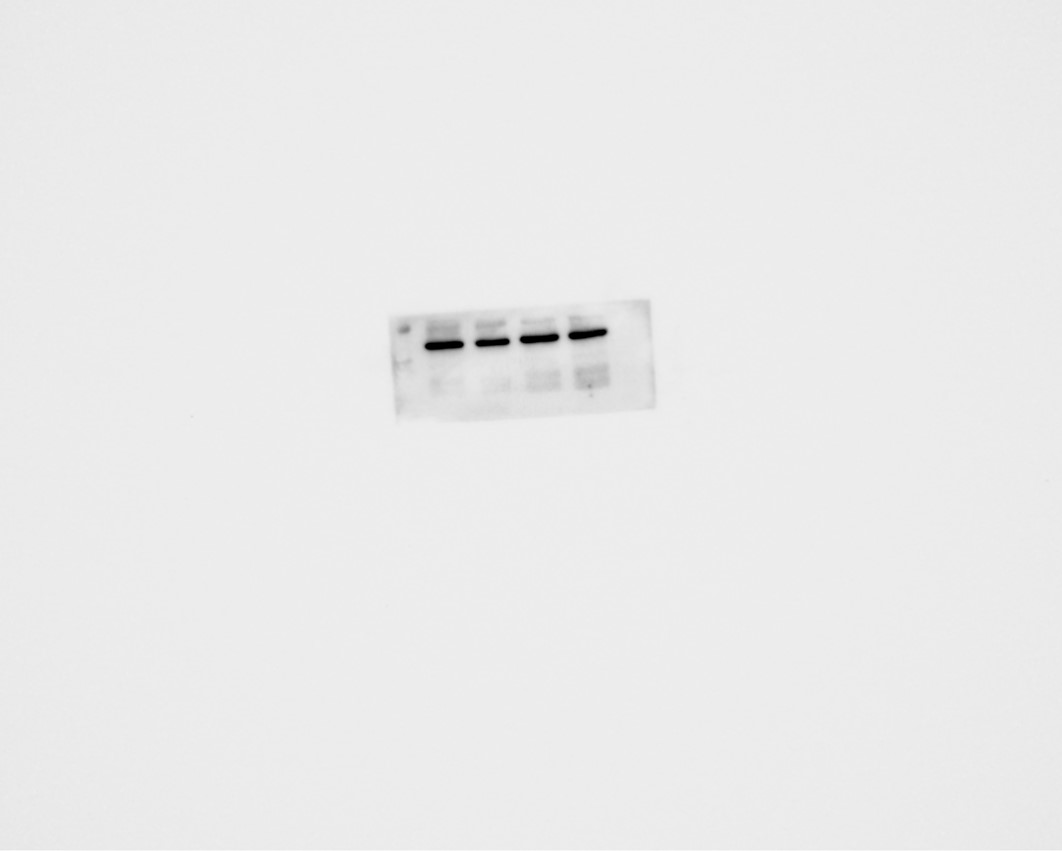

Supplement: Supplementary file 1 [file DataSheet1.ZIP › Orginal Images of Western Blot/Figure 4/4.jpg]

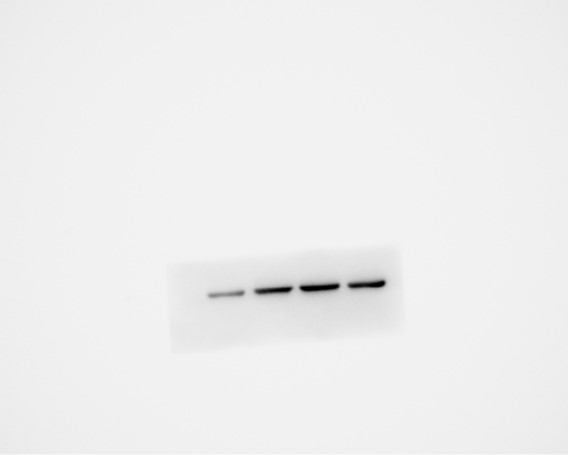

Supplement: Supplementary file 1 [file DataSheet1.ZIP › Orginal Images of Western Blot/Figure 5/1.jpg]

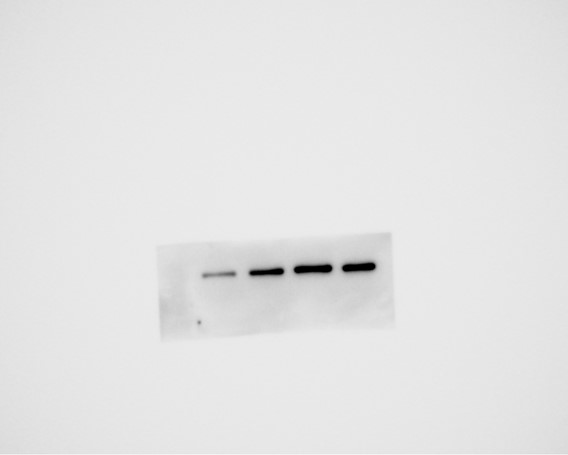

Supplement: Supplementary file 1 [file DataSheet1.ZIP › Orginal Images of Western Blot/Figure 5/2.jpg]

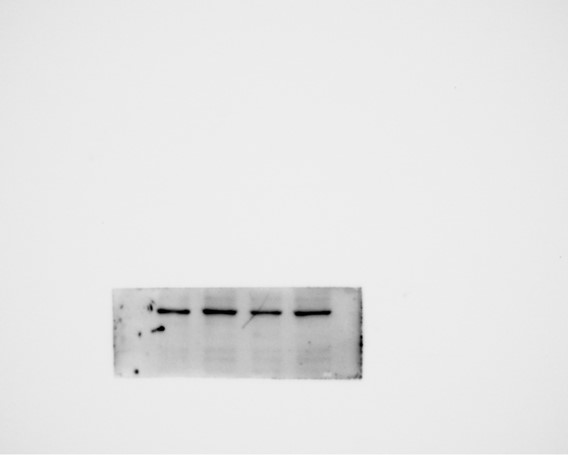

Supplement: Supplementary file 1 [file DataSheet1.ZIP › Orginal Images of Western Blot/Figure 5/3.jpg]

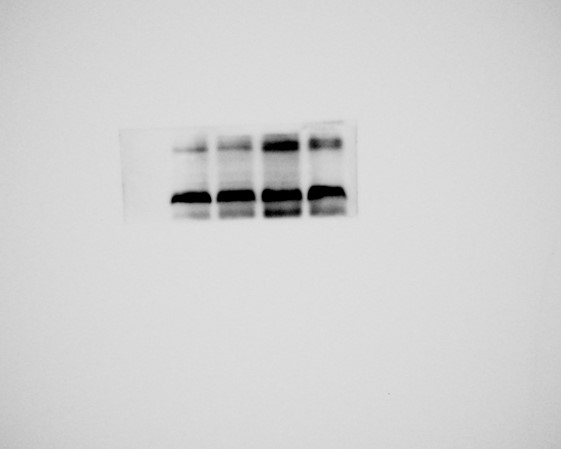

Supplement: Supplementary file 1 [file DataSheet1.ZIP › Orginal Images of Western Blot/Figure 5/4.jpg]

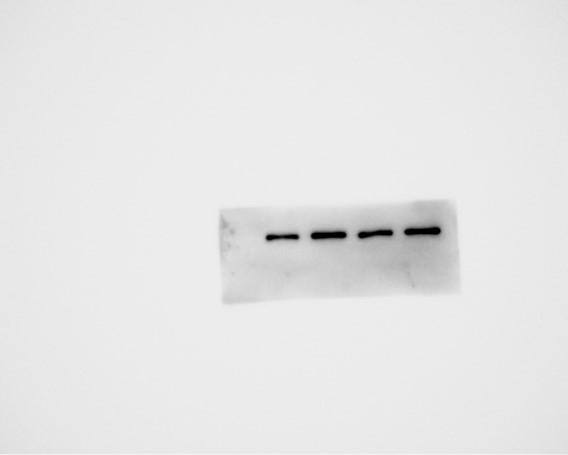

Supplement: Supplementary file 1 [file DataSheet1.ZIP › Orginal Images of Western Blot/Figure 5/5.jpg]

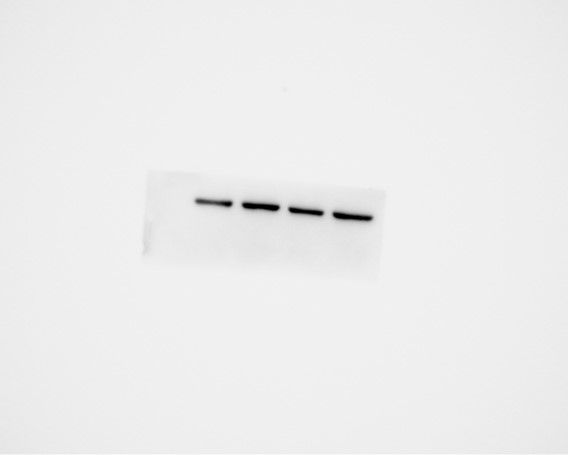

Supplement: Supplementary file 1 [file DataSheet1.ZIP › Orginal Images of Western Blot/Figure 5/6.jpg]

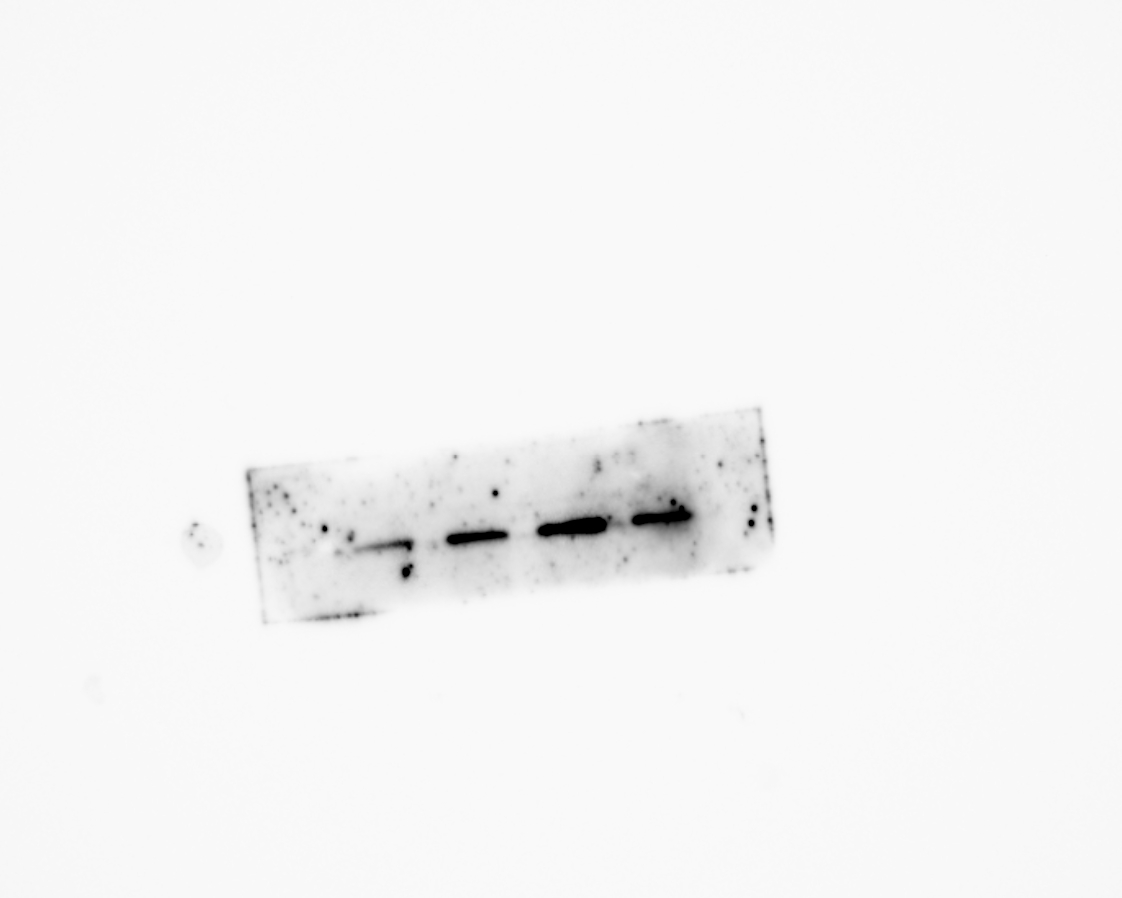

Supplement: Supplementary file 1 [file DataSheet1.ZIP › Orginal Images of Western Blot/Figure 5/7.jpg]

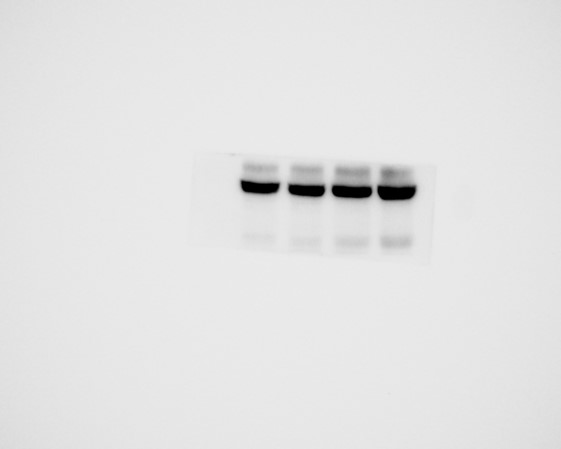

Supplement: Supplementary file 1 [file DataSheet1.ZIP › Orginal Images of Western Blot/Figure 5/8.jpg]

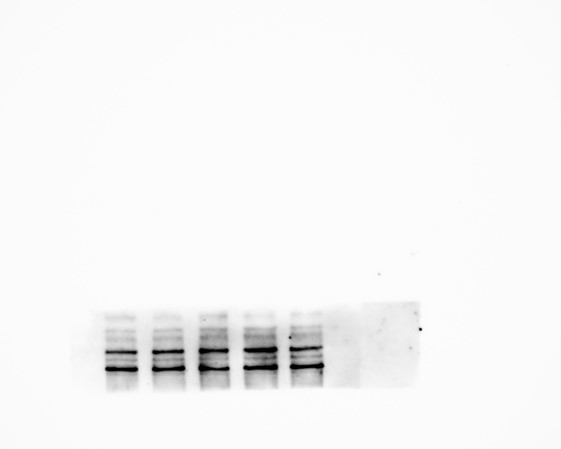

Supplement: Supplementary file 1 [file DataSheet1.ZIP › Orginal Images of Western Blot/Figure 6/1.jpg]

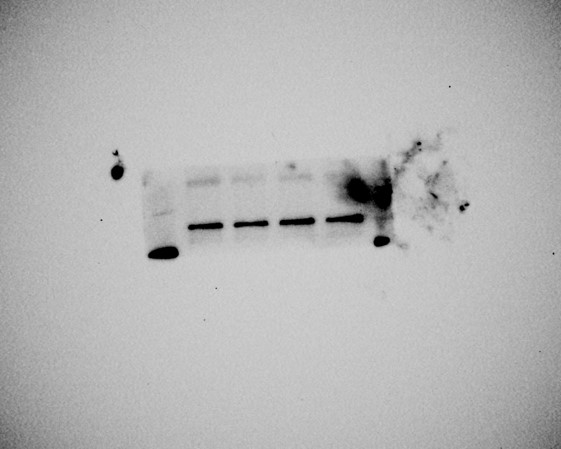

Supplement: Supplementary file 1 [file DataSheet1.ZIP › Orginal Images of Western Blot/Figure 6/10.jpg]

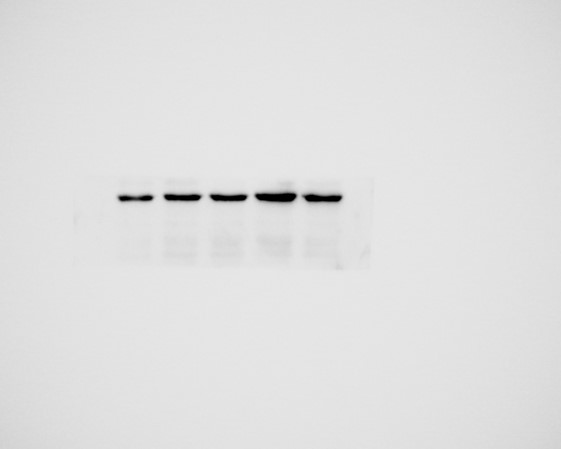

Supplement: Supplementary file 1 [file DataSheet1.ZIP › Orginal Images of Western Blot/Figure 6/2.jpg]

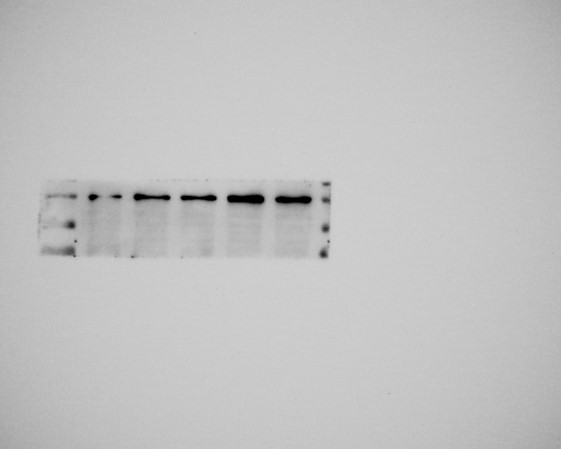

Supplement: Supplementary file 1 [file DataSheet1.ZIP › Orginal Images of Western Blot/Figure 6/3.jpg]

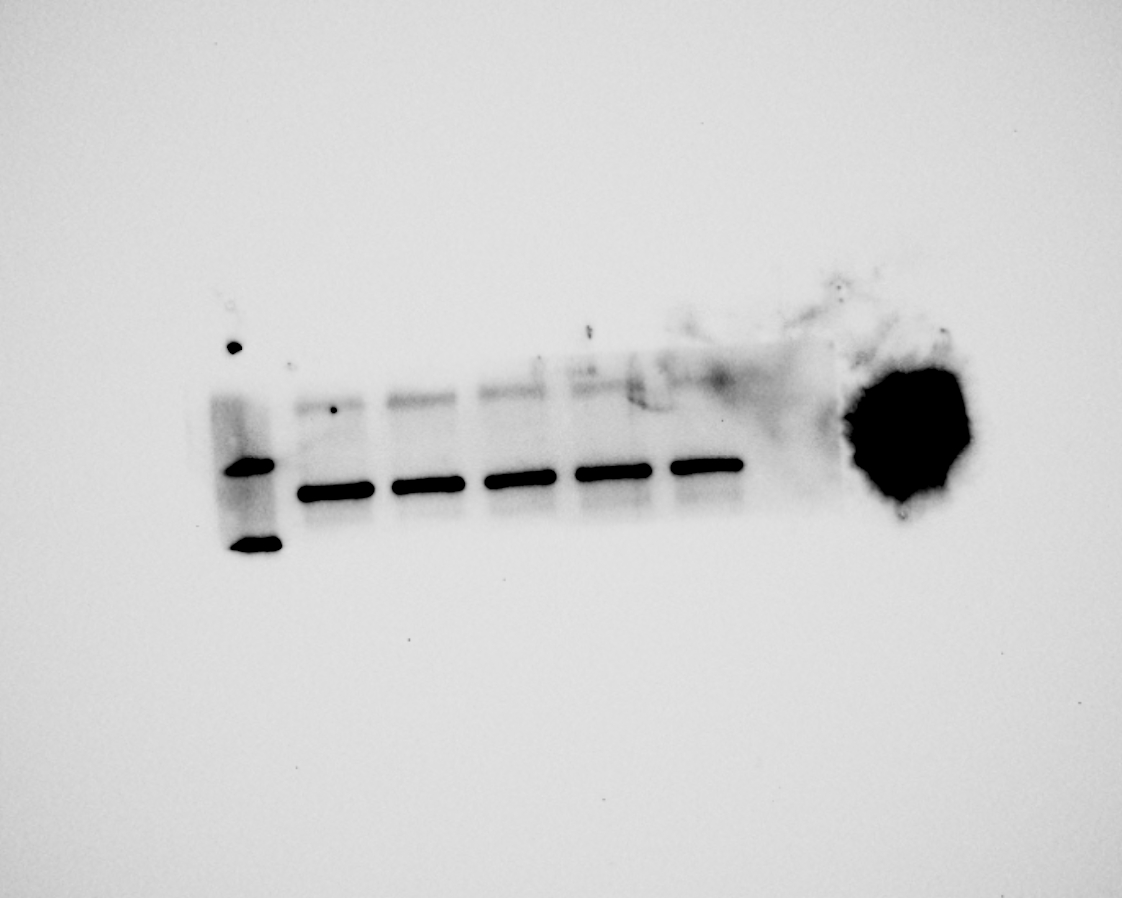

Supplement: Supplementary file 1 [file DataSheet1.ZIP › Orginal Images of Western Blot/Figure 6/4.jpg]

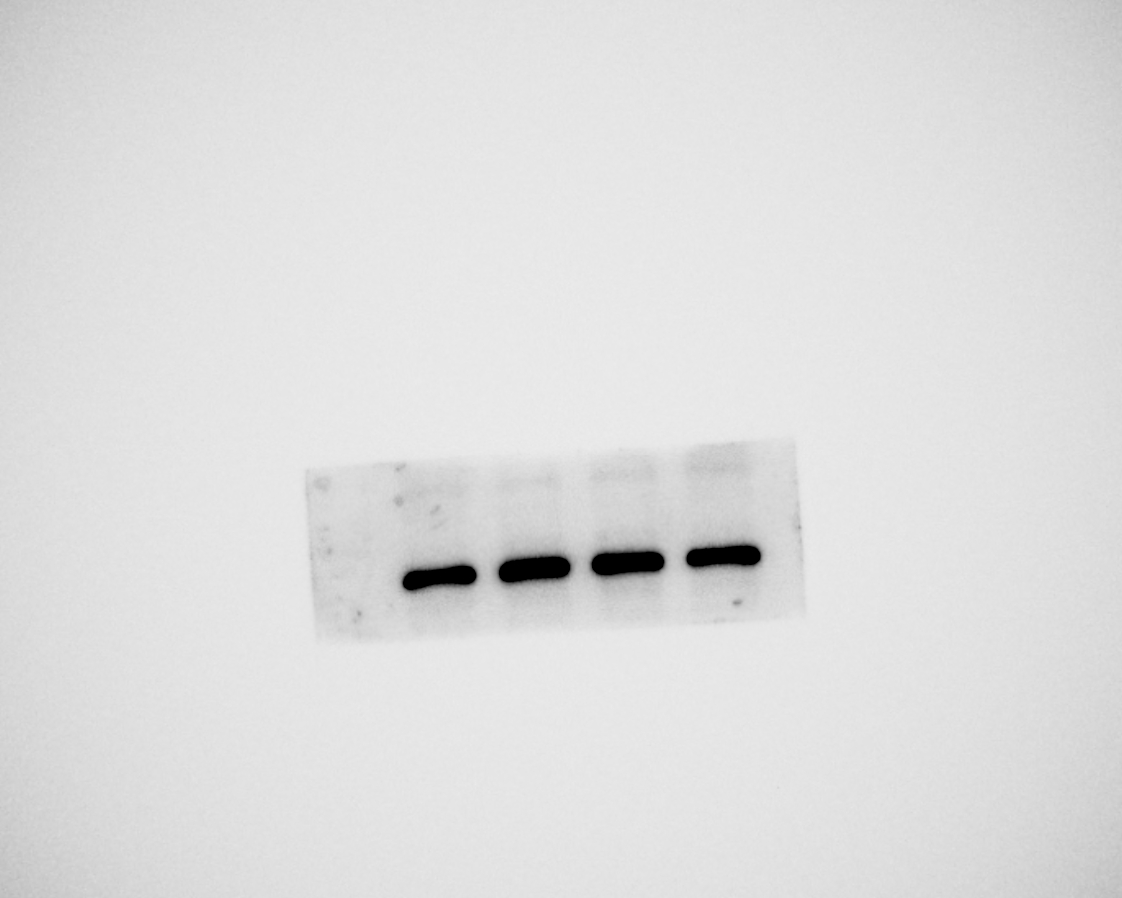

Supplement: Supplementary file 1 [file DataSheet1.ZIP › Orginal Images of Western Blot/Figure 6/5.jpg]

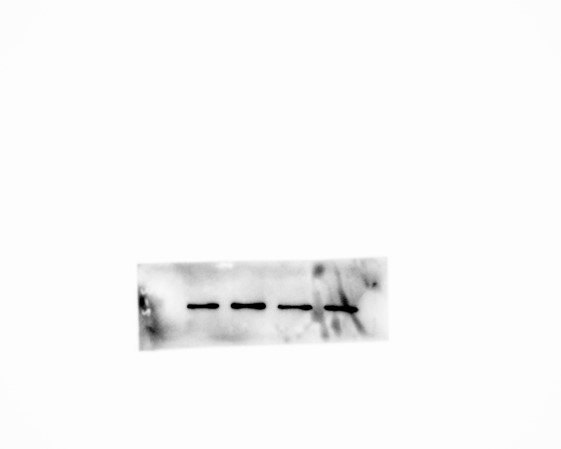

Supplement: Supplementary file 1 [file DataSheet1.ZIP › Orginal Images of Western Blot/Figure 6/6.jpg]

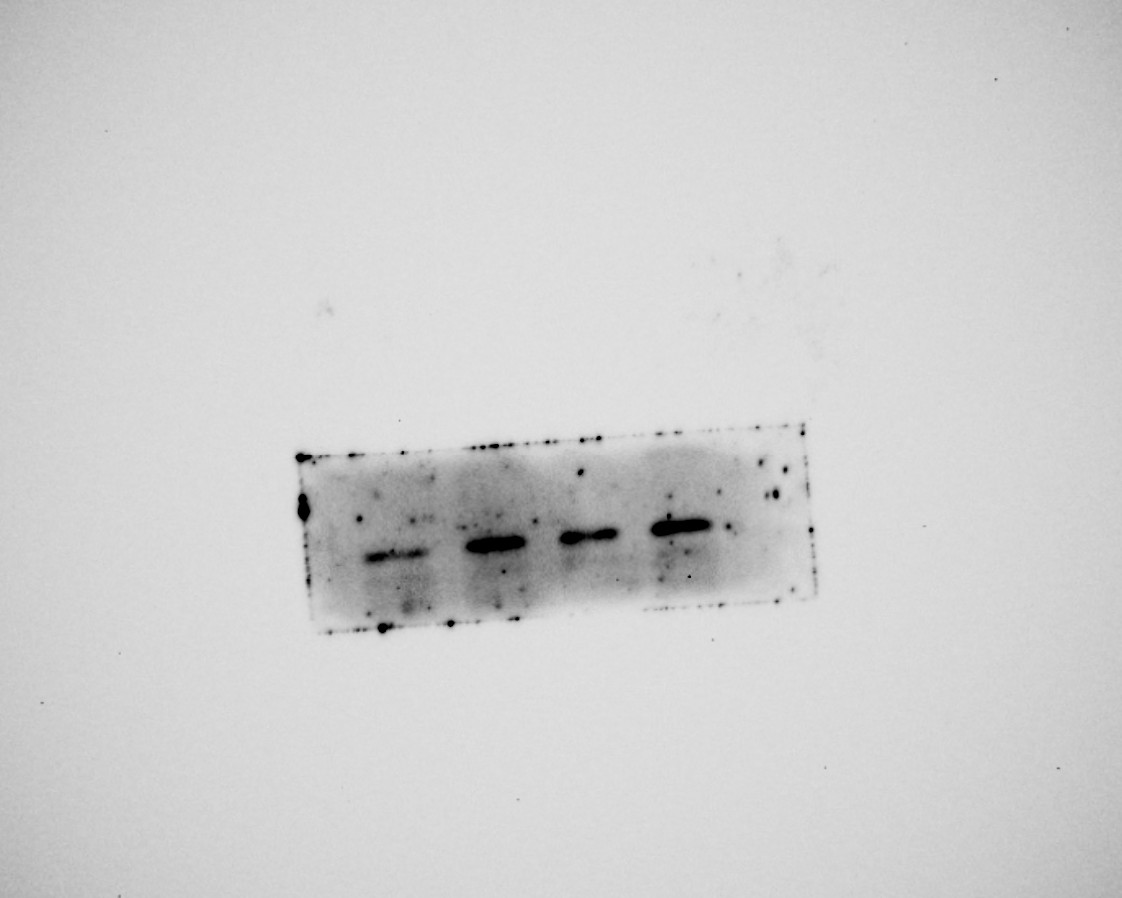

Supplement: Supplementary file 1 [file DataSheet1.ZIP › Orginal Images of Western Blot/Figure 6/7.jpg]

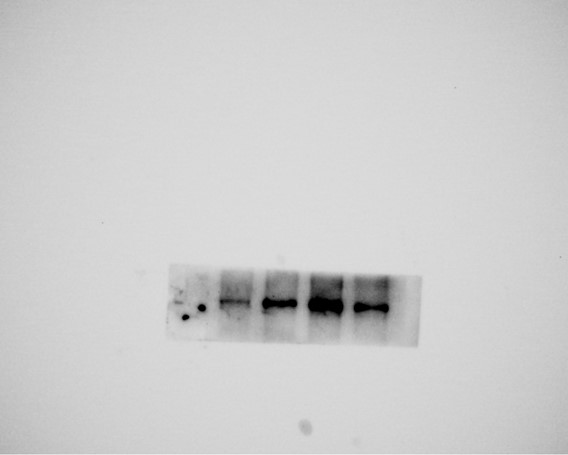

Supplement: Supplementary file 1 [file DataSheet1.ZIP › Orginal Images of Western Blot/Figure 6/8.jpg]

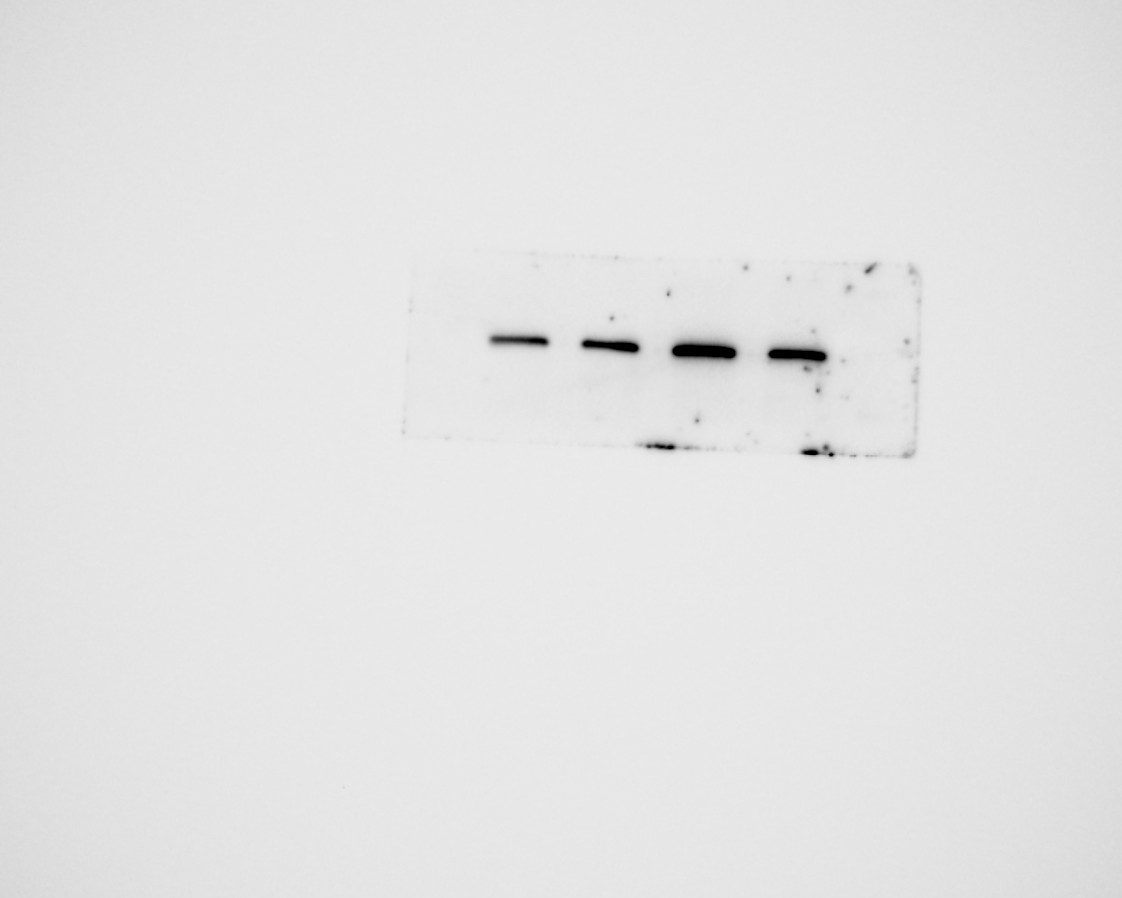

Supplement: Supplementary file 1 [file DataSheet1.ZIP › Orginal Images of Western Blot/Figure 6/9.jpg]
